# In Situ Polymerization Controlled Growth of Perovskite via Benzoic Acid Crosslinking Agent for Highly Efficient and Mechanically Robust Flexible Perovskite Solar Cell

**DOI:** 10.1002/advs.202508253

**Published:** 2025-07-21

**Authors:** Yankun Yang, Na Yang, Chenxi Zhang, Shiqi Li, Yang Hao, Qinjun Sun, Zhihui Chen, Shengzhong (Frank) Liu, Fei Guo, Jianfeng Lu, Yuying Hao

**Affiliations:** ^1^ College of Physics and Optoelectronics Engineering Shanxi Key Lab of Photovoltaic Technology and Application Key Lab of Advanced Transducers and Intelligent Control System Taiyuan University of Technology Taiyuan 030024 China; ^2^ Key Laboratory of Applied Surface and Colloid Chemistry National Ministry of Education Shaanxi Engineering Lab for Advanced Energy Technology School of Materials Science and Engineering Shaanxi Normal University Xi'an 710119 China; ^3^ Institute of New Energy Technology College of Physics & Optoelectronic Engineering Jinan University Guangzhou 510632 China; ^4^ State Key Laboratory of Silicate Materials for Architectures Wuhan University of Technology Wuhan 430070 China

**Keywords:** benzoic acid crosslinker, flexible perovskite solar cell, perovskite solar cell

## Abstract

The controlled growth of perovskite on flexible substrates is essential for achieving highly efficient and stable flexible perovskite solar cells (FPSCs). Herein, a novel strategy of 4‐hydroxybenzoic acid (4‐HBA) is developed to regulate the crystallization dynamics of perovskite. In situ cross‐linking network of 4‐HBA provided an excellent formwork for high‐quality perovskite growth by controlling excessive nucleation and prolonging crystallization. Grain boundary defects, residual stress, and Young's modulus of the perovskite film are greatly decreased. The dynamic hydrogen bonds between free hydroxyl groups and carboxyl groups/halogen enhanced the self‐healing ability of perovskite film. The electrostatic interaction between the benzene ring of 4‐HBA and uncoordinated Pb²⁺, coupled with π‐π stacking of 4‐HBA, enhanced the conductivity of perovskite film. As a result, the optimal power conversion efficiency (PCE) of rigid perovskite solar cells (PSCs) and FPSCs reached 24.76% and 22.73%, respectively. The FPSCs retained 91% of initial efficiency after 10 000 bending cycles at 5 mm radius. The FPSCs after 4000 bends at 2 mm radius, regained 89% original PCE with 60℃ heating. The unencapsulated FPSCs retained 83% original PCE after 1000 h of Air Mass 1.5 Global illumination. This work offered an efficient strategy for high‐efficiency and robust FPSCs.

## Graphical abstract

This study proposes a novel strategy to improve the performance of perovskite solar cells through the incorporation of the crosslinking agent 4‐HBA into the perovskite layer. The multifunctional properties of 4‐HBA contribute to power conversion efficiencies of 24.76% for rigid devices and 22.73% for flexible devices, both exhibiting remarkable stability.

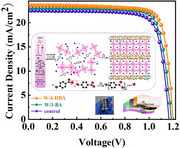

## 1 Introduction

Flexible perovskite solar cells (FPSCs) have garnered widespread attention due to their advantages of high efficiency, excellent flexibility, high power‐to‐weight ratio, and low‐temperature processability.^[^ ^1^, ^2^ ^]^ At present, the power conversion efficiency (PCE) of FPSCs has exceeded 25%, which is considered as a promising candidate for some distinctive applications, such as wearable and portable electronics, vehicle and building integrated photovoltaics etc.^[^ ^3^, ^4^ ^]^ Despite the remarkable achievements in the performance of FPSCs, there still exist notable problems and challenges. One of the great challenges is the deposition of high‐quality perovskite films on flexible substrates due to the coarse surface, the low temperature tolerance, and the low thermal conductivity of the plastic substrate.^[^ ^5^, ^6^, ^7^ ^]^ Furthermore, the high Young's modulus of polycrystalline perovskite leads to the accumulation of residual stress at grain boundaries and thus makes the film fragile and vulnerable under mechanical deformation.^[^ ^8^, ^9^ ^]^ Besides, the erosion of perovskite by water and oxygen in the air results in perovskite degradation, and thus poses an irreversibly negative impact on the stability of FPSCs.^[^ ^10^, ^11^ ^]^ Therefore, reducing defects and releasing stress at grain boundaries of perovskite and enabling effective isolation of water/oxygen are very crucial for enhancing the efficiency and stability of FPSCs.

Recently, the crosslinker strategies have been developed to improve the performance and stability of FPSCs. The crosslink monomers can form a 3D network structure through chemical or physical interactions under certain conditions.^[^ ^12^ ^]^ Consequently, the crosslinking agent can serve as a scaffold to interconnect perovskite grain boundaries, potentially addressing the issues of excessive grain boundary defects, high Young's modulus, and inadequate environmental stability of perovskite. For instance, Wu et al. utilized the small molecule cross‐linking agent of bis ((3‐methylchloroethyl) methyl) thiophen‐2,5‐ dicarboxylate (OETC) to limit the number of perovskite nucleation sites and reduce grain boundary defects, thus maintaining 93% of the original efficiency after 5000 bending cycles.^[^ ^5^ ^]^ However, small‐molecule crosslinked scaffolds are not unstable and brittle and thus can not maintain the long‐term stability of FPCS under harsh operating conditions.^[^ ^13^, ^14^ ^]^ Lan et al. employed a self‐healing organic elastomer of polyurethane to decrease the Young's modulus of perovskite and alleviate the residual stress in perovskite, thereby significantly enhancing the mechanical stability of the device.^[^ ^15^ ^]^ However, self‐healing of dynamic covalent bonds often requires external conditions, such as high temperatures or prolonged exposure to ultraviolet light or oxygen, which can induce additional lattice strains or phase transitions that adversely affect the performance and stability of perovskite films.^[^ ^16^, ^17^ ^]^ Xue et al. adopted a macromolecular long‐chain crosslinking agent (PEGDMA) to regulate effectively crystallization and reduce grain boundary defects through in situ crosslinking and anchor chemically at grain boundaries.^[^ ^18^ ^]^ Moreover, PEGDMA effectively releases residual strain, allowing the device to maintain more than 86% of its initial PCE after 5000 bending cycles under a bending radius of 5 mm. But the introduction of long‐chain polymer into perovskite easily invokes the electrical insulation effect in the photoactive layer.^[^ ^5^, ^18^ ^]^ Consequently, selecting the appropriate crosslinking agents, not only to address the existing issues of perovskites but also not to trigger negative impacts and entanglements, is very imperative for the realization of efficient and stable FPSCs.

In this work, we proposed a novel crosslinking agent strategy of 4‐hydroxybenzoic acid (4‐HBA) to modify FPSCs. It was identified that 4‐hydroxybenzoic acid (4‐HBA) with a hydroxyl group and carboxyl group underwent intermolecular crosslinking through esterification reactions under the mild conditions, and thus held promise for addressing the aforementioned challenges.^[^ ^19^ ^]^ The binding of carbon‐oxygen double bond (C ═ O) in 4‐HBA to Pb^2+^ imposed restrictions on the number of perovskite nucleation sites and thus modulated the crystal growth of perovskite and reducing grain boundary defects. The 4‐HBA with different crosslinking degrees facilitated hydrogen bonding interactions between free Hydroxyl group (‐OH), carboxyl groups, and halogen anions, along with intermolecular hydrogen bonding interactions of the double helix 4‐HBA.^[^ ^20^ ^]^ These interactions not only decreased the Young's modulus and the residual stress of perovskite films but also compensated for the structural weaknesses associated with small‐molecule crosslinking agents. The dynamic hydrogen bonds exhibited rapid binding in response to the mild stimulation and thus favored the self‐healing of perovskite films. Moreover, the electrostatic interaction between the benzene ring in 4‐HBA and uncoordinated Pb^2+^ ions in perovskite, coupled with π‐π stacking of 4‐HBA, enhanced the electrical conductivity of the perovskite film by providing channels of charge extraction and transport, solving the entanglement of electrical insulation of macromolecular polymers. In addition, the hydrophobic nature of 4‐HBA improved the environmental stability of perovskite.^[^ ^21^ ^]^ Benefited from the abovementioned advantages, the optimal PCE of 24.76% for the 4‐HBA‐modified rigid PSCs and the optimal PCE of 22.73% for the corresponding FPSCs were achieved. The 4‐HBA‐modified FPSCs still maintained 91% of the original efficiency after 10 000 bending cycles with a bending radius of 5 mm. The FPSCs, after 4000 bending cycles at a radius of 2 mm were able to recover 89% of the original efficiency when treated at 60 °C and then stored in a nitrogen environment for 24 h, demonstrating a strong self‐healing ability. Besides, the unencapsulated FPSCs with 4‐HBA maintained 82% of the original efficiency after being stored for 1600 h in an air environment with RH 20%–30% and temperature of 25–30 °C, and maintained 83% of the original efficiency under the AM1.5G light irradiation for 1000 h. In general, this work provided a very efficient strategy for the attainment of highly efficient and robust FPSCs.

## 2 Results and Discussion

Initially, the crosslinking mechanism of 4‐HBA was investigated. To examine the different effects of crosslinkers and general small molecules on PSCs, 4‐HBA and 3‐benzoylpropionic acid (3‐BA) were introduced into the perovskite precursor at a concentration of 0.4 mg mL^−1^, respectively. It is worth noting that 3‐BA has a similar molecular structure as 4‐HBA, but lacks crosslinking function (Figure S1, Supporting Information). To investigate the cross‐linking reaction of 4‐HBA, the macroscopic observations were made by capturing the photos of 4‐HBA before and after the crosslinking reaction at 100 °C for 30 min, as detailed in Figure S2 (Supporting Information), which provides compelling evidence for the cross‐linking behavior of 4‐HBA.^[^ ^22^ ^]^ Subsequently, the cross‐linking mechanism of 4‐HBA was further clarified through Fourier transform infrared (FTIR) analysis, as illustrated in Figure S3 (Supporting Information). The results showed that the carbon‐oxygen single bond (C─O) stretching vibration peaks shifted from 1207 to 1205 cm^−1^ and from 1050 to 1042 cm^−1^. The stretching vibrational peak corresponding to the C ═ O of the carboxyl group shifted from 1715 to 1723 cm⁻¹. Meanwhile, the carbon‐carbon single bond (C‐C) peak associated with the benzene ring shifted from 861cm^−1^ to 851cm^−1^. All these indicate that 4‐HBA undergoes the cross‐linking reaction. The possible cross‐linking pathway of 4‐HBA was inferred as an esterification reaction. It was also observed that the ‐OH stretching vibration peak was weakened and shifted from 3337 cm^−1^ to 3255 cm^−1^ in the cross‐linked 4‐HBA, suggesting that 4‐HBA underwent cross‐linking and the cross‐linked network of 4‐HBA formed by intermolecular hydrogen bonds.^[^ ^23^, ^24^ ^]^

To more intuitively determine the crosslinking degree of 4‐HBA, the nuclear magnetic hydrogen spectra (^1^H‐NMR) of the cross‐linked 4‐HBA were tested, and the reaction degree was roughly determined by the integral peak area ratio. As shown in (Figure S4, Supporting Information), after polymerization treatment, the signal corresponding to hydrogen in the ─OH group shifted from 4.58 to 4.53 ppm.^[^ ^25^ ^]^ This downfield shift in the chemical shift originated from the influence of intermolecular hydrogen bonding interactions of 4‐HBA. The integral areas of the hydrogen signals from the ‐OH and ‐COOH groups exhibited a distinct decrease, indicating the polymerization of 4‐HBA, as shown in Table S1 (Supporting Information). By analyzing the integral area ratio of the consumed ‐OH groups to the total ‐OH groups in the monomer, the reaction degree was measured to be ≈27%, as shown in Table S2 (Supporting Information). These findings further support that 4‐HBA underwent crosslinking reaction through esterification reactions. Besides, the matrix‐assisted laser desorption/ionization time‐of‐flight mass spectrometry (MALDI‐TOF) was used to further study the polymerization degree of 4‐HBA. The MALDI‐TOF spectra of 4‐HBA are presented in Figure S5 (Supporting Information). The spectra of the oligomers are represented mainly by peaks of protonated molecules [M+H]^+^, [M+Na]^+^, and [M+K]^+^. The peaks corresponding to the molecular weight were summarized in Table S3 and Figure S5 (Supporting Information). The results indicated that the chain lengths formed by polymerization ranged from 2 to 13. The average degree of polymerization of 4‐HBA is identified as 7.

**Figure 1:**
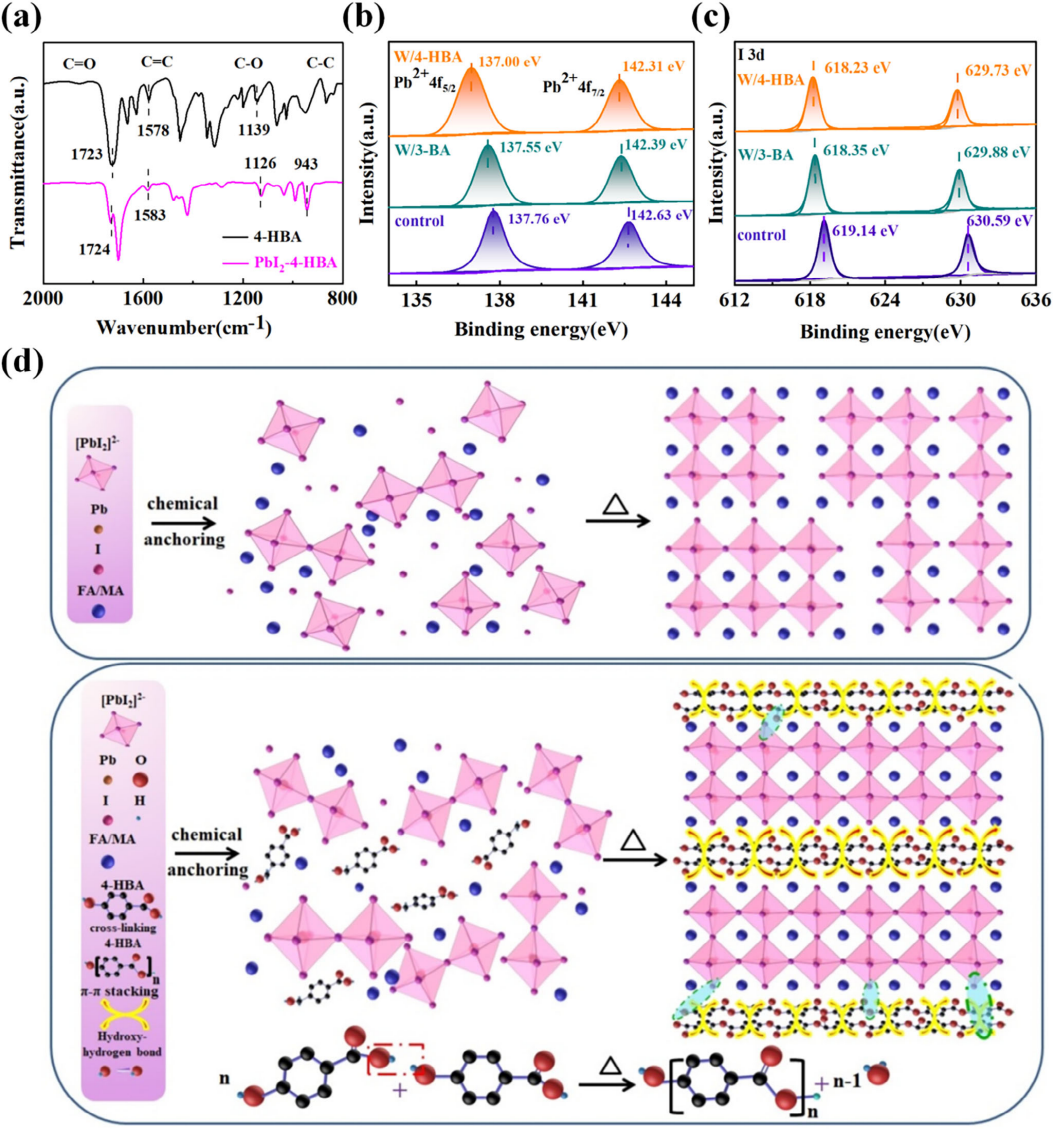
a) FTIR spectra of the pristine 4‐HBA and the 4‐HBA‐doped PbI_2._ b) Pb4f and c) I3d XPS spectra of the pristine PbI_2_ and 3‐BA or 4‐HBA‐doped PbI_2_ films. d) Schematic diagram of the interaction mechanism between 4‐HBA and perovskite.

Afterward, the interaction between 4‐HBA and perovskite was investigated by means of FTIR spectra and X‐ray photoelectron spectroscopy (XPS) tests. When 4‐HBA was doped in PbI_2_, the stretching vibration peak of C ═ O bond in FTIR spectra shifted from 1723 to 1701 cm^−1^, and the stretching vibration peak of C─O shifted from 1139 to 1126 cm^−1^, as illustrated in **Figure** **1**a. This result was related to the coordination interaction between the C ═ O/C─O groups in 4‐HBA and the Pb^2+^ ions.^[^ ^5^, ^26^ ^]^ Simultaneously, the carbon‐carbon double bond (C ═ C) peaks of the benzene ring in 4‐HBA shifted from 1578cm^−1^ and 1583cm^−1^, indicating that the Pb^2+^ ions had electrostatic interaction with the benzene ring.^[^ ^27^ ^]^

Figure 1b presents the XPS spectra of the pristine PbI_2_ and the 4‐HBA and 3‐BA‐modified PbI_2_. It can be found that for the PbI_2_ doped with 4‐HBA, the two main peaks of Pb4f moved from 137.76 to 137.00 eV and from 142.63 to 142.31 eV, respectively, further reflecting that the coordination interaction between the C ═ O/C─O of 4‐HBA and the Pb^2+^ ions formed by sharing the lone‐pair electron of the oxygen atom.^[^ ^28^ ^]^ The results also showed that the coordination interaction of PbI_2_ with 4‐HBA was stronger than that with 3‐BA. Additionally, it was found that upon the incorporation of 4‐HBA, the I3d spectra also exhibited the shift from 619.14 and 630.59 eV to 618.23 and 629.73 eV, as shown in Figure 1c. This observation suggested the formation of the hydrogen bonding interactions between I^−^ ions and hydrogen atoms of hydroxyl groups.^[^ ^29^ ^]^ To further understand the interactions between 3‐BA, 4‐HBA, and perovskite, we conducted the theoretical analysis based on DFT computation, as illustrated in Figure S6 and Table S4 (Supporting Information). The results indicated that the binding energy of 4‐HBA with perovskite (11.69 eV) far exceeds that of 3‐BA with perovskite (4.57 eV), demonstrating a significantly stronger interaction due to the anchoring of 4‐HBA to the perovskite via double coordination bonds and hydrogen bonds. Whereas 3‐BA was anchored to the perovskite via a single coordination bond. Based on the above‐mentioned characterizations, the principal diagram of the interaction between 4‐HBA and perovskite was proposed, as shown in Figure 1d. 4‐HBA interacted with perovskite through the coordination bonds between the C ═ O/C─O groups and the Pb^2+^ ions, the hydrogen bonds between ─OH groups and I^−^ ions, and the electrostatic interaction between the benzene ring and Pb^2+^ ions, and hence it was expected to regulate the perovskite crystallization and passivate the perovskite defects. The abundant hydrogen bonds in the cross‐linking network of 4‐HBA could endow the high flexibility and self‐healing ability of the perovskite film when the bending and stretching stresses were applied.^[^ ^30^, ^31^ ^]^

**Figure 2:**
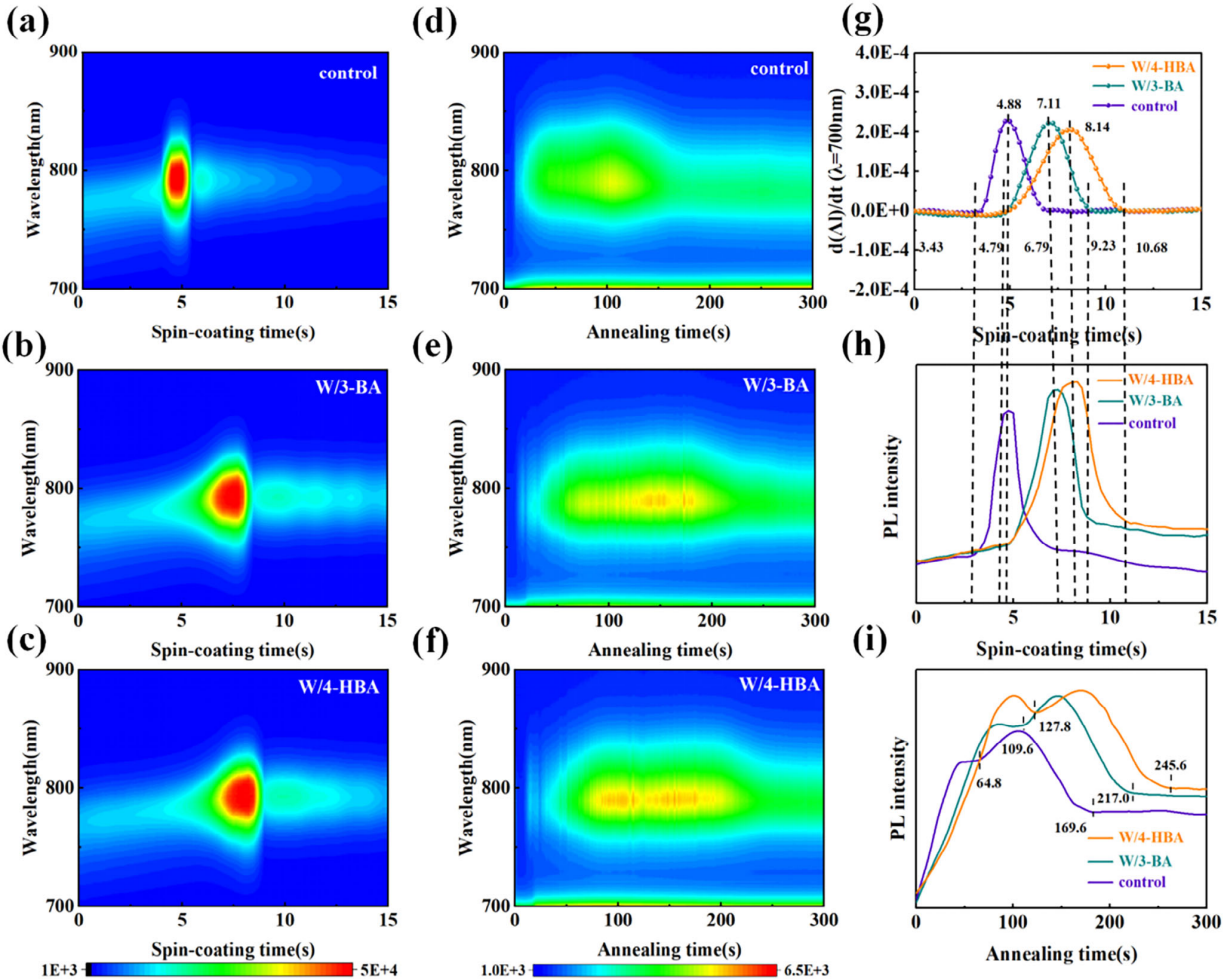
a–c) In situ PL spectra during spin‐coating organic ammonium salt on PbI_2_ film. d–f) In situ PL spectra of perovskite wet films during annealing. g) The differentiation of the evolution curves of absorption intensity with time at 700 nm from Figure S4 (Supporting Information). h) PL intensity evolution curves with time at 790 nm extracted from Figure 2a–c. i) PL intensity evolution curves with time extracted from Figure 2d–f.

Next, the impact of the crosslinked 4‐HBA on the nucleation and crystallization behavior of perovskite was systematically investigated. The spin‐coating process of organic ammonium salt solution on PbI_2_ films was monitored in real‐time using in situ absorption spectroscopy (Figure S7, Supporting Information) and in situ photoluminescence (PL) spectroscopy (**Figure** **2**a–c). To analyze this process in detail, the curves of absorption intensity evolution with time at λ = 700 nm were extracted from (Figure S8, Supporting Information) and differentiated (Figure 2g). Meanwhile, the PL intensity evolution with time at 790 nm was extracted from Figure 2h. By analysis, the three stages of the formation of the solvent complex, the rapid and slow formation and growth of perovskite microcrystals, were distinguished. In the first stage, the organic ammonium salt solution permeated into the underlying PbI_2_ film, and the polyiodine compounds formed, in which six coordination sites of Pb^2+^ were occupied by organic solvents or/and I^−^. Correspondingly, the dAI/dt values were near zero with dynamic fluctuation due to the thin film interference.^[^ ^32^ ^]^ It was found that the formation process of the solvent complex was more significantly prolonged from 3.43 to 4.79 s by doping 4‐HBA or 3‐BA due to the entanglement of 4‐HBA or 3‐BA.

In the following stage, the dAI/dt value showed a rapid increase, meaning a rapid nucleation and growth process when the supersaturated solution was achieved with solvent volatilize. The Pb^2+^ coordination sites occupied by the organic solvent were replaced by the organic ammonium salt rapidly and the solvent complex was converted into perovskite microcrystals. Meanwhile, the corresponding PL intensity at 790 nm extracted from Figure 2a–c also showed a sharp upward trend (Figure 2h), reflecting the rapid formation and free growth of a large number of perovskite microcrystals. In addition, it was observed that this stage was also prolonged from 1.45 s of the pristine film to 2.32 s of the 3‐BA doped film and 3.35 s of the 4‐HBA doped film. This was beneficial for the formation of perovskite microcrystals with high crystallinity, which was also proved by the strongest PL intensity of the 4‐HBA doped sample. Subsequently, the slow growth stage of perovskite microcrystals began, in which the value of dAI/dt showed a decreasing trend until it reached zero, indicating that the formation and growth rate of perovskite microcrystals gradually decreased because the exchange reaction became slow with the continuous consumption of solvent molecules. Meanwhile, it was found that the corresponding PL intensity also showed a rapid decline, which was possible due to the fact that the crystals grew up gradually and then contacted each other to form grain boundaries. The defects at continually increased grain boundaries caused PL intensity to decrease continuously. It is also likely that the redissolution of the formed perovskite microcrystals by residual solvent caused PL quenching. It is found that this stage was also obviously lengthened from 1.90 s for the control sample to 2.12 and 2.54 s for the 3‐BA and 4‐HBA doped samples, respectively. It should be attributed to the reason that the stronger coordination interaction between 4‐HBA and Pb^2+^ delayed the exchange reaction and the conversion from solvent complex to perovskite phase.

Next, the nucleation and growth process of perovskite during the annealing was also detected by in situ PL spectra, as shown in Figure 2d–f. In order to analyze this process distinctly, the PL peak intensity evolution with time at 790 nm and the PL spectra at 10 s of annealing were extracted from the in situ PL spectra, as shown in Figures 2i and S9 (Support Information), in which two stages can be distinguished. At the first stage, the PL intensity shows a rapid upward trend. Under high temperature, the solvent in wet films evaporated rapidly, and the supersaturated solution formed again, the nucleation and growth of perovskite occurred subsequently, which led to the rapid increase of PL intensity.^[^ ^33^, ^34^ ^]^ It was found that the rise rate PL intensity was slowest among the three samples, which was attributed to the stronger coordination interaction between 4‐HBA and Pb^2+,^ limiting the nucleation sites of perovskite and delaying the conversion of perovskite phase. As a result, the crystallinity of perovskite was well improved, and thus, the perovskite containing 4‐HBA exhibited the highest PL intensity. According to the abovementioned investigation, the cross‐linking network structure of 4‐HBA should form during the annealing, which provides a more favorable template for regulating perovskite crystal growth. Therefore, 4‐HBA was more effective than 3‐BA with similar function groups in regulating the nucleation and growth of perovskite. The second stage presented the dissolution and recrystallization process of perovskite microcrystals, i.e., the Ostwald ripening process.^[^ ^35^, ^36^ ^]^ As the buried solvent further volatilizes, the tiny microcrystals are dissolved, and then re‐nucleate and grow on the surface of large grains, finally fusing to form mature perovskite crystal grains.^[^ ^37^ ^]^ In this stage, the PL intensity showed an upward trend again due to the fusion growth of perovskite grains in the secondary crystallization process. As the perovskite crystals grew continuously and met with other crystals to form new grain boundaries, leading to the increase of defects and hence the decline of PL intensity. It was found that the recrystallization time of perovskite containing 4‐HBA was 117.8 s, while those of the doped 3‐BA and pristine samples were 107.4 and 104.8 s, respectively. These results indicated that 4‐HBA also prolonged the recrystallization process of perovskite. This could originate from the fact that the cross‐linking network of 4‐HBA decreased the nucleation sites of perovskite, so that perovskite microcrystals had more growth spaces, and thus promoted its sustainable growth. The PL peak intensity of recrystallization perovskite containing 4‐HBA was strongest, and there was a slight blue shift (Figure S9, Support Information), implying that the grain boundaries of 4‐HBA‐modified perovskite films were the least, and the defect state density was lowest, which was mainly attributed to the cross‐linking network of 4‐HBA enabling the high‐quality perovskite crystal. This should also be related to the defect passivation effect due to the stronger chemical interaction of 4‐HBA with perovskite relative to 3‐BA.

**Figure 3:**
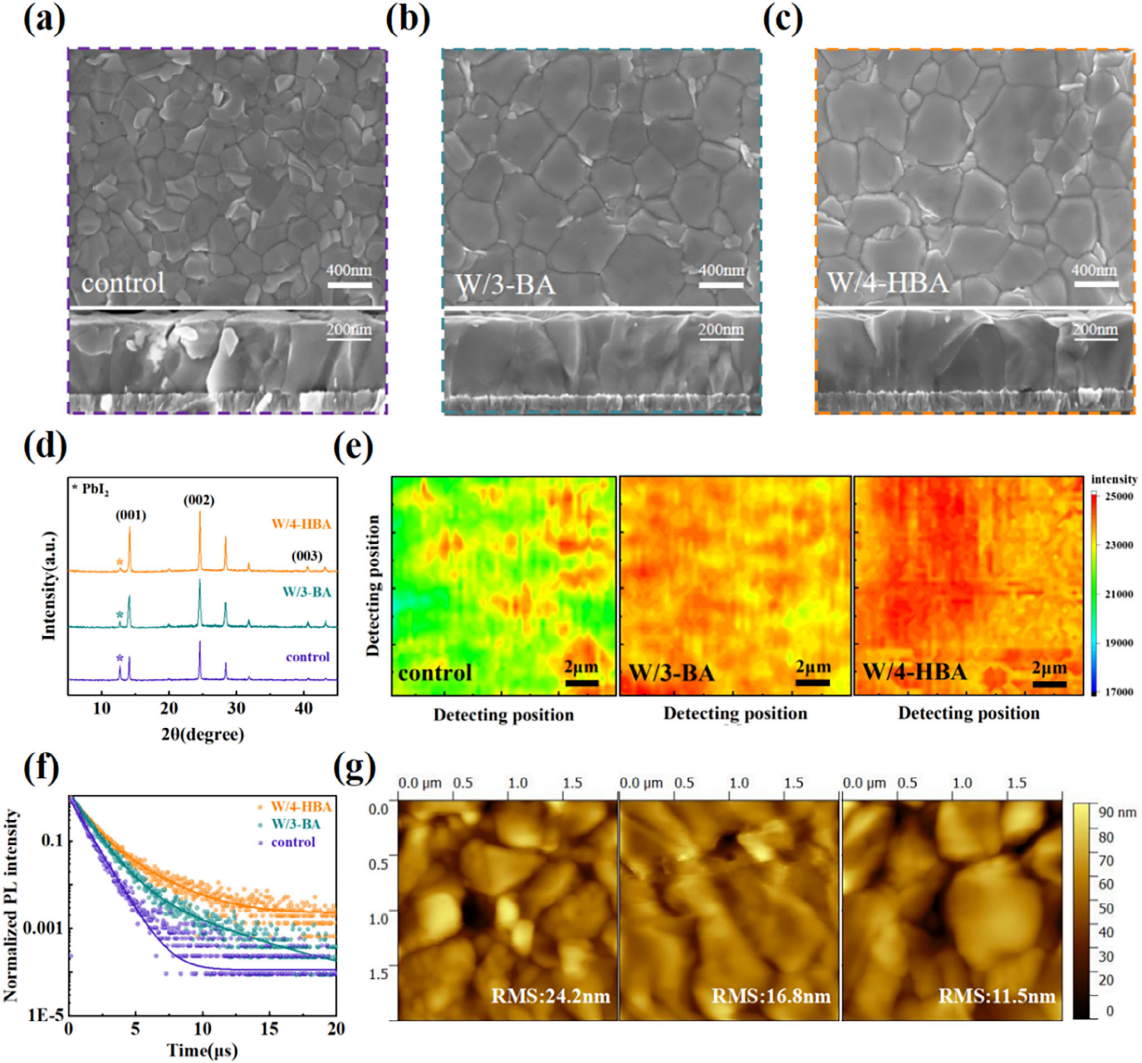
a–c) Top surface and cross‐section SEM images, d) XRD patterns, e) PL‐mapping, f) transient PL spectra, and g) AFM images of different perovskite films.

To further confirm the beneficial impact of 4‐HBA on the perovskite crystallization kinetics process, the morphology of perovskite films was investigated by scanning electron microscopy (SEM). **Figure** **3**a–c shows the surface and cross‐section morphologies of the different perovskite films. According to the grain size statistics, the average grain size of 4‐HBA‐modified perovskite film increased to 937 nm from 584 nm of the pristine film, which was also larger than the 873 nm of the 3‐BA‐modified perovskite. The cross‐sectional SEM images also displayed that the 4‐HBA modified film was occupied by dense, uniform, and film‐through large grains, obviously exceeding the 3‐BA‐modified films, especially the pristine film. These results proved that the cross‐linking 4‐HBA was more useful for controlling the crystallization of perovskite by reducing the nucleation number and prolonging the nucleation and crystal growth process during weather spin‐coating or annealing, and hence more effectively reduced the defect state density of perovskite films.

The crystallinity quality of the perovskite films was further investigated by X‐ray diffraction (XRD), as detailed in Figure 3d. It can be seen that the diffraction peaks of 4‐HBA‐modified perovskite film were significantly stronger and the residues of PbI_2_ were the least, proving that 4‐HBA not only induced a better‐crystallized perovskite film but also facilitated the complete conversion from PbI_2_ to perovskite.^[^ ^38^ ^]^

The crystallinity and uniformity of perovskite films were also checked by photoluminescence (PL) mapping test, as illustrated in Figure 3e. This test provided precise insights into PL intensity distribution across the surface of perovskite films. The results indicated that the incorporation of 4‐HBA led to a more uniform and stronger fluorescence distribution. In comparison, the PL strength and uniformity of the pristine and 3‐BA‐modified films were obviously lower. These findings were consistent with the SEM and XRD results, being attributed to the better‐crystallized perovskite film and the passivation effect of 4‐HBA, which mitigated the defect states in perovskite films and suppressed non‐radiative recombination. This conclusion was also confirmed by the longer carrier lifetime of 4‐HBA‐modified perovskite films (Figure 3f; Table S5, Supporting Information). Furthermore, atomic force microscopy (AFM) tests were used to characterize the uniformity and roughness of films, as detailed in Figure 3g. The RMS of the pristine and 3‐BA‐modified perovskite films were 24.2 and 16.8 nm, respectively, while that of 4‐HBA‐modified perovskite films was dropped to 11.5 nm. The smoother surface was very beneficial for reducing the interfacial non‐radiative recombination and enhancing interfacial contact with transport layer.^[^ ^39^, ^40^ ^]^

**Figure 4:**
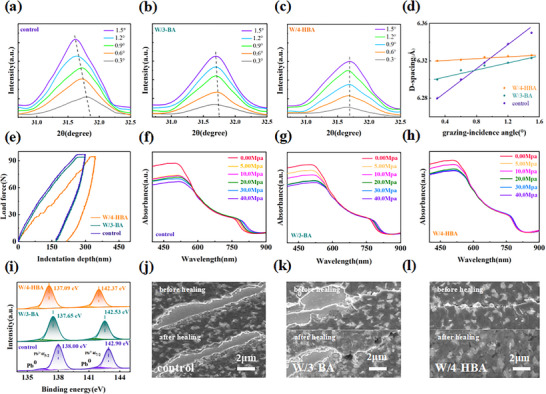
a–c) Grazing incidence XRD patterns and d) *d*‐spacing values from perovskite (012) crystal planes at different grazing incidence angles. e) Nanoindentation curves of different perovskite films. f–h) Variation of UV absorption spectra under different pressures for different perovskite films. i) Pb 4f XPS spectra of different perovskite films after bending of 2000 cycles under the bending radius of 2 mm. j–l) SEM images before and after self‐healing for different perovskite films that undergoing bending of 4000 cycles at the bending radius of 2 mm.

In addition to the roles of regulating perovskite crystallization and passivating perovskite defects, 4‐HBA was also expected to improve the flexibility of the perovskite film. To characterize the mechanical properties of perovskite films, the stress distribution of perovskite films at different depths was studied by grazing‐incidence X‐ray diffraction (GIXRD) tests at the different grazing incidence angles (*ω*) of 0.3°, 0.6°, 0.9°, 1.2°, and 1.5°, as illustrated in **Figure** **4**a–c. By comparison, it was found that the diffraction peaks associated with the (012) crystal planes moved to the smaller 2*θ* with the change of *ω* from 0.3° to 1.5° for both the pristine and the 3‐BA‐modified perovskite films, whereas that of 4‐HBA‐modified perovskite films did not change with the increase of grazing incidence angle. This implied that the lattice expansion was inhibited and the residual stress was alleviated in the perovskite layer by the cross‐linking 4‐HBA.^[^ ^41^, ^42^ ^]^ Furthermore, the lattice spacing *d* of perovskite at different grazing incidence angles was calculated based on GIXRD results, as shown in Figure 4d. It was clear that the residual tensile stress was more effectively alleviated by 4‐HBA than 3‐BA, which should be attributed to the crosslinked scaffold of 4‐HBA and the more effective defect passivation effect of 4‐HBA.^[^ ^43^, ^44^ ^]^

The Young's modulus characterization was performed by means of nanoindentation, as shown in Figure 4e. The Young's modulus of the 4‐HBA‐modified perovskite film was 25.05 GPa, which was significantly lower than that of the pristine film (40.19 GPa) and the film modified with 3‐BA (31.85 GPa). The results showed that 4‐HBA increased the flexibility of the perovskite film, which originated from the hybrid cross‐linking network of 4‐HBA as a bridge connecting perovskite grains and releasing the lattice stress of perovskite.

In order to confirm the increased flexibility of 4‐HBA‐modified perovskite film, the pressure resilience tests were carried out by measuring the variation of UV absorption spectra of perovskite films subjected to diverse pressures, as shown in Figure 4f–h. The pristine and 3‐BA‐modified perovskite films exhibited a distinct red shift in band‐edge of absorption with a substantial decrease in absorption intensity as the applied pressure intensified, while the perovskite film modified with 4‐HBA crosslinker had the least decline in absorption intensity and only a slight red shift in band‐edge of absorption. The same phenomenon also appeared in steady‐state PL spectra under different pressures (Figure S10, Supporting Information). These results indicated that the crystal lattice deformation of perovskite with the increase of pressure was inhibited by the cross‐linking network structure of 4‐HBA due to the stronger coordination bond and richer hydrogen bond interactions.^[^ ^45^ ^]^ Although 3‐BA also alleviated the pressure‐induced crystal lattice deformation to a certain extent, it cannot provide an adequate compression resistance for perovskite.

In addition, the bending stability of the different perovskite films was compared and analyzed. X‐ray photoelectron spectroscopy (XPS) of perovskite films on flexible substrate before and after bending of 2000 cycles under the bending radius of 2 mm was tested, as illustrated in Figure 4i. The results showed that after bending, the two main Pb4f peaks of perovskite underwent mechanical degradation with the shifts of 0.24/0.27 eV and 0.10/0.14 eV for the pristine and 3‐BA‐modified perovskite films. While the Pb 4f peaks of the perovskite film containing 4‐HBA shifted only 0.09 and 0.06 eV. The results suggested that the hybrid cross‐linked networks of 4‐HBA mitigated the mechanical degradation of perovskite films by energy dissipation during mechanical deformation due to the presence of dynamic hydrogen bonds and electrostatic interactions between the benzene ring and Pb^2+^ ions.^[^ ^24^ ^]^ The bending stability of perovskite films was also characterized intuitively by SEM tests (Figure 4j–l). After the bending of 4000 cycles under the bending radius of 2 mm, serious cracks appeared in the pristine and the 3‐BA modified perovskite films, while the perovskite with 4‐HBA had only slight cracks. After being treated at 60 °C for 1 h and placed in the glove box for 24 h, the cracks of the perovskite films modified with 4‐HBA were healed. This was attributed to the rich, dynamic non‐covalent bonds in the 4‐HBA included perovskite. These bonds had the ability to bind rapidly under mild stimulation, and hence endowed the excellent self‐healing ability of the perovskite film.^[^ ^46^, ^47^, ^48^ ^]^

**Figure 5:**
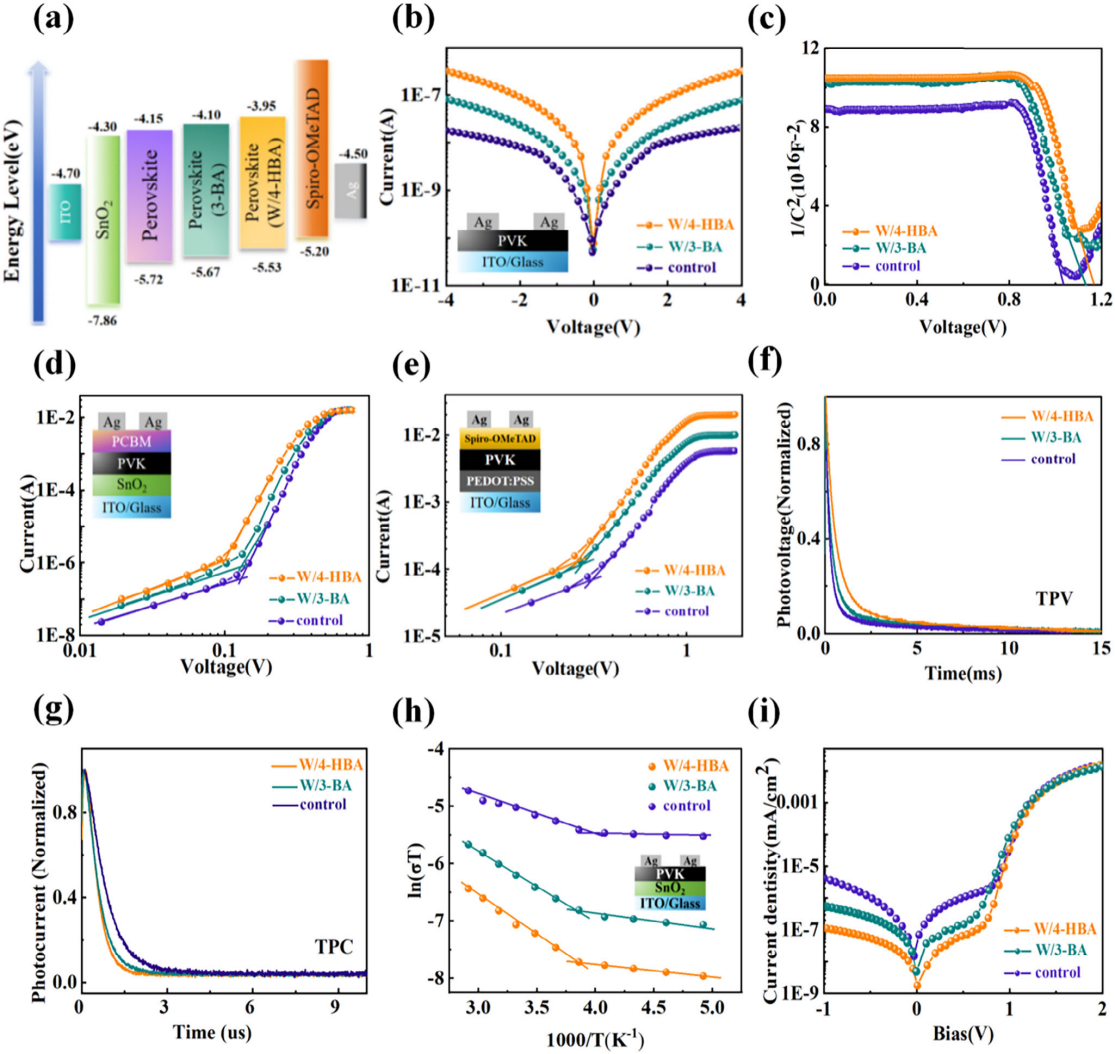
a) Energy level arrangement diagram of PSCs. b) Transverse conductivity of different perovskite films. c) Mott–Schottky diagram of PSCs. d) log*I*‐log*V* curve of single‐electron device. e) log*I*‐log*V* curve of single‐hole device. f)Transient optical voltage (TPV) curves of PSCs. g) Transient photocurrent (TPC) curves of PSCs. h) Conductivity change with temperature under dark conditions. i) Dark state *J*‐*V* curves of PSCs.

In addition to the mechanical properties of perovskite films, the energy level arrangement is very important for carrier extraction and transport. Therefore, the ultraviolet photoelectron spectroscopy (UPS) was used to investigate the energy band structure of perovskite films, as illustrated in Figure S11, (Supporting Information). Combined with the Tauc plots of different perovskite films (Figure S12, Supporting Information), the detailed energy level information of different perovskite films was obtained, as shown in **Figure** **5**a. It is observed that the conduction and valence bands of the 4‐HBA‐modified perovskite film shifted upward, indicating that 4‐HBA made the energy level arrangement more matched at the interface of perovskite/hole transport layer, which was expected to promote hole extraction and reduce interfacial recombination losses.^[^ ^49^ ^]^

To assess the effect of cross‐linking 4‐HBA on the electrical properties of perovskites, the longitudinal and transverse conductivity measurements of perovskite films were conducted, as shown in Figure S13 (Supporting Information) and Figure 5b. The results indicated that the longitudinal conductivity of 4‐HBA‐modified perovskite film was efficiently increased. Specifically, the longitudinal conductivity of 4‐HBA modified perovskite films was 9.3 × 10^−5^ S cm^−1^, being higher than 7.1 × 10^−5^ S cm^−1^ of the pristine films and 8.4 × 10^−5^ S cm^−1^ of the 3‐BA‐modified film, respectively. To further understand the effects of 3‐BA and 4‐HBA on the electrical conductivity of perovskite, the intermolecular *π*‐*π* interactions for 3‐BA and 4‐HBA molecules were investigated by DFT computation. The six original configurations with *π*‐*π* interaction were built in the same modes for 3‐BA and 4‐HBA molecules, respectively, and their stable molecular configurations were obtained (Figure S14, Supporting Information). Based on the above molecular configuration, the intermolecular π‐π interaction energies for 4‐HBA and 3‐BA molecules were quantitatively evaluated (Table S6, Supporting Information). It can be found that the intermolecular *π*‐*π* interaction of 4‐HBA is significantly stronger than that in 3‐BA, which provides more effective charge extraction and transport channels, and thus solves the electrical insulation entanglement of common polymer additives.^[^ ^50^ ^]^

Next, C‐V measurements were performed to characterize the built‐in potential (V_bi_) of PSCs, as illustrated in Figure 5c. It was shown that *V*_bi_ of PSCs with 3‐BA and 4‐HBA modification was increased from 1.132 to 1.165, and 1.173 V, respectively, which should be associated with the matched interface energy level arrangement and the reduced defect states. The increased *V*_bi_ would provide a stronger driving force for charge separation and transfer, thereby being expected to improve the collection of charge carriers and thus increase the open circuit voltage (*V*oc) and fill factor (FF) of PSCs.

The reduction of defect state densities in perovskite films was one of the main reasons for the improved electrical properties of devices. To quantitatively study the defect state density (*N*_t_) of perovskite films, the single‐electron and single‐hole devices were assembled, and *J*‐*V* curves of them were tested, as shown in Figure 5d,e. The first corner point of the ohmic region and the (TFL) region represents the defect filling limit voltage (*V*_TFL_). The single‐electron devices with 3‐BA and 4‐HBA had the reduced *V*_TFL_ from 0.132 to 0.128 V and 0.105 V, respectively. Based on the formula of *N*_t_ = *V*_TFL_ *ε*_0_ *ε*_r_/*eL* ^2^, where *e* is the elementary charge, *ɛ*_r_ is the material dielectric constant, *ɛ*_0_ is the vacuum dielectric constant, and *L* is the film thickness, the electron trap density values were calculated. The results indicated that *N*_t_ of the devices based on 3‐BA and 4‐HBA decreased from 2.045×10^15^ to 1.983×10^15^ and 1.626 × 10^15^ cm^−3^. Similarly, the *V*_TFL_ of single‐hole devices with 3‐BA and 4‐HBA was reduced from 0.135 to 0.112 V and 0.101 V, respectively. Consequently, *N*_t_ of 3‐BA and 4‐HBA modified devices also decreased from 2.091 × 10^15^ cm^−3^ to 1.983 × 10^15^ cm^−3^ and 1.627 × 10^15^ cm^−3^. Compared with the pristine and the 3‐BA‐modified devices, 4‐HBA devices had the significantly lowest carrier trapping state density due to the improved perovskite crystallinity and defect passivation, and hence were expected to enhance carrier mobility and suppress non‐radiative recombination.

Afterward, the transient photovoltage (TPV) and transient photocurrent (TPC) of PSCs were tested to research carrier recombination and transport process within the scope of microseconds to milliseconds, as detailed in Figure 5f,g. According to the TPV curves, it was found that the carrier lifetime of the device with 4‐HBA modification was significantly increased by 3.34 and 5.22 ms compared with the pristine and the 3‐BA modified devices. The relatively long carrier lifetime meant that the carrier non‐radiative recombination was mitigated by 4‐HBA. The TPC curves demonstrated that the 4‐HBA‐optimized devices had a faster photocurrent decay of 0.44 µs than 0.75 µs and 0.69 µs of the printine and the 3‐BA‐optimized devices, suggesting more efficient charge transfer and extraction.

Subsequently, the activation energy (*E*a) of ion migration was calculated based on the temperature‐dependent conductivity under dark conditions, as shown in Figure 5h. By the equation of *σ*(*T*) = (*σ*_0_/*T*)exp(‐*E*_a_/*k*_B_ *T*), where *σ*_0_ is a constant, *k*_B_ is Boltzmann's constant, *σ* is the ionic conductivity, *T* is the temperature, the *E*a of 0.065 eV was obtained for the 4‐HBA modified devices, which was higher than 0.034 and 0.053 eV of the pristine and 3‐BA‐modified devices, indicating that the ion migration in perovskite films was alleviated due to the reduced defect state density and the released residual stress in 4‐HBA modified film. The negligible zero‐drift and the lower leakage current density in the dark *J*−*V* curve of 4‐HBA‐modified PSCs compared to those of the pristine and the 3‐BA‐modified PSCs, also indicated that the introduction of 4‐HBA inhibited ion migration and reduced defect state density (Figure 5i).

**Figure 6:**
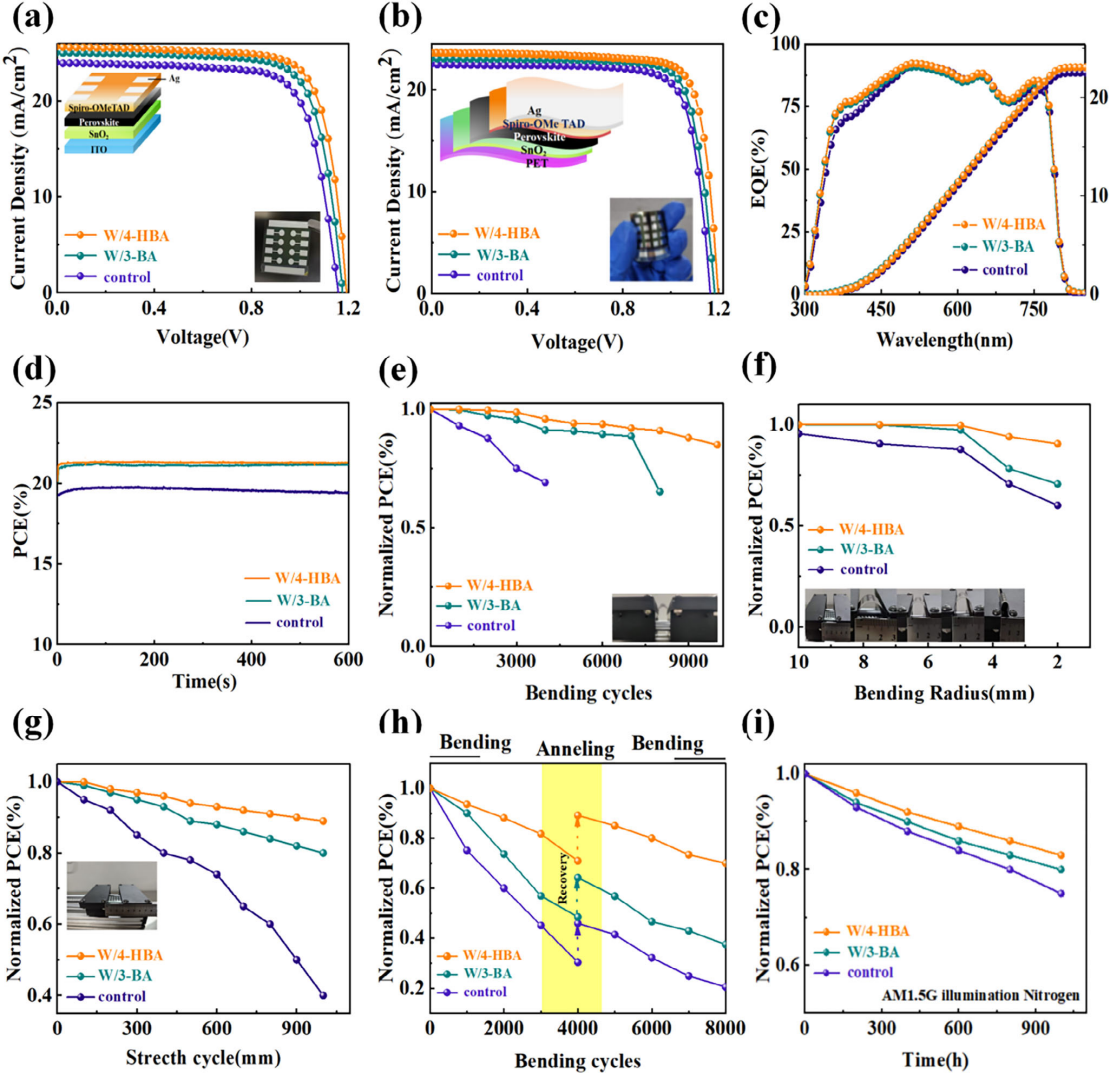
a) *J*‐*V* curve of rigid PSCs. b) *J*‐*V* curve of FPSCs test. c) External quantum efficiency (EQE) of FPSCs. d) Maximum power output stability of FPSCs. e) Efficiency evolution of FPSCs with bending cycle times at a bending radius of 5 mm. f) Efficiency change of FPSCs with bending radii under a bending cycle of 2000 times. g) Efficiency evolution of FPSCs with stretching‐cycle times. h) Efficiency recovery of FPSCs after self‐healing. i) Efficiency statistics of FPSCs under continous light irradiation.

Based on the different perovskite films, we prepared the rigid PSCs with the device structure of glass/ITO/SnO_2_/perovskite /PEAI/Spiro‐OMeTAD/Ag. And the optimized doping concentration of 0.4 mg ml^−1^ was achieved for both 4‐HBA and 3‐BA additives by investigating the influence of different doping concentrations of 4‐HBA and 3‐BA on the photovoltaic performance of the devices (Table S7, Supporting Information). The *J*‐*V* curves of champion devices for each kind were shown in **Figure** **6**a. The best PCE of the 4‐HBA‐modified PSCs was 24.76% with *J*_SC_ of 25.77 mA cm^−^^2^, *V*_OC_ of 1.19 V, and FF of 0.80, being evidently higher than 22.32% and 23.83% of the pristine and the 3‐BA‐modified PSCs. Based on the photovoltaic parameter statistics of 30 PSCs (Figure S15, Supporting Information), it was found that the 4‐HBA‐modified PSCs also exhibited more excellent photovoltaic performance and repeatability compared with the pristine and the 3‐BA‐modified PSCs.

Based on the different perovskite films, the FPSCs with the structure of PET/SnO_2_/perovskite/Spiro‐OMeTAD/PETA/Ag were also prepared. The *J*‐*V* curves of the optimal devices for each kind were illustrated in Figure 6b. Likewise, the 4‐HBA‐modified FPSCs exhibited the highest PCE of 22.73% with *J*_SC_ of 23.63 mA cm^−^^2^, *V*oc of 1.20 V, and FF of 0.80, which exceeded the PCEs of 20.69% and 21.78% of the pristine and the 3‐BA‐modified PSCs. The EQE tests (Figure 6c) also showed the reliability of the *J*‐*V* results. According to the statistical analysis of photovoltaic parameters from 30 FPSCs, as shown in Figure S16 and Table S8 (Supporting Information), all photovoltaic parameters of the 4‐HBA‐modified FPSCs exhibited significant improvement with commendable repeatability. In addition, the maximum power output tests of FPSCs for 600 s, as shown in Figure 6d indicated that the 4‐HBA‐modified devices maintained a higher and more stable output than the pristine and the 3‐BA‐modified devices.

Subsequently, the flexural and tensile stability of FPSCs was investigated in detail. The results indicated that the 4‐HBA‐optimized FPSC was able to maintain 91% of its original efficiency after 10 000 cycles of bending at a radius of 5 mm. In contrast, the pristine and 3‐BA‐modified FPSCs only endured the bending of 4000 and 8000 cycles, as illustrated in Figure 6e. Even undergoing 2000 bending cycles at a radius of 2 mm, the 4‐HBA‐modified FPSC also retained 90% of its initial efficiency, while the pristine and 3‐BA‐modified FPSCs sustained just 60% and 70% of their original efficiency, as shown in Figure 6f. Simultaneously, the tensile cyclic stress tests of FPSCs were performed, as detailed in Figure 6g. When the 4‐HBA‐modified FPSC was stretched by 10%, it still maintained 93% of its original efficiency, while the pristine and 3‐BA‐modified FPSCs maintained only 37% and 85% of the original efficiency. The self‐healing ability of the 4‐HBA‐modified FPSC after undergoing the bending of 4000 cycles at a radius of 2 mm was also checked. Through self‐healing efficiency statistics (Figure 6h), it can be seen that the flexible device after self‐healing treatment recovered the original efficiency of 89%. Compared with the common polymer 3‐BA, the 4‐HBA crosslinker exhibited a stronger self‐healing ability due to the dynamic hydrogen bond interaction of 4‐HBA crosslinking agent.

In addition to the mechanical stability of FPSCs, the environmental, thermal, and light stability are also crucial. The efficiency evolution of the unencapsulated device stored for 1600 h in air with RH 20%‐30% and temperature of 25–30 °C as well as the water contact angle of perovskite films, were shown in (Figure S17, Supporting Information). The efficiency statistics showed that the FPSCs modified with 4‐HBA still maintained 82% of the original efficiency, which was more stable than the pristine and 3‐BA modified devices. This was attributed to the environmental stability of 4‐HBA modified perovskite film (Figure S18, Supporting Information). The 4‐HBA cross‐linking network improved the hydrophobicity of the perovskite film and thus achieved the effective isolation of water and oxygen. Then the thermal stability of the devices was assessed, as shown in (Figure S19, Supporting Information). The results indicated that the unencapsulated FPSCs 4‐HBA‐modification retained 79% of the initial efficiency stored in nitrogen for 320 h at 60 °C, obviously surpassing the other devices. Additionally, the light stability tests in Figure 6i revealed that the 4‐HBA‐modified FPSCs maintained 83% of the original efficiency under Air Mass 1.5 Global (AM1.5G) continuous irradiation for 1000 h, also exceeding the other devices. The above characterizations revealed that the 4‐HBA cross‐linking agent enabled higher moisture‐, heat‐, and light‐resistant FPSCs.

## 3 Summarize

In conclusion, we proposed a strengthening and self‐healing strategy based 4‐HBA crosslinker for attaining highly efficient and robust FPSCs. The crosslinking network 4‐HBA limited the excessive nucleation of perovskite and prolonged the crystal growth of perovskite, and thus enabled the high crystallization of perovskite films with obviously fewer grain boundary defects and lower grain lattice stress, and smaller Young's modulus by 62.3% relative to the pristine perovskite films. The rich dynamic hydrogen bonds between free ‐OH and carboxyl groups/halogen anions endowed the high self‐healing ability of perovskite films. The electrostatic interaction between the benzene ring in 4‐HBA and uncoordinated Pb^2+^ ions in perovskite, coupled with π‐π stacking of 4‐HBA, enhanced the electrical conductivity of the perovskite film. As a result, the optimal efficiencies of 24.76% and 22.73% were realized for rigid PSCs and FPSCs with 4‐HBA‐modification, respectively. Moreover, 4‐HBA considerably improved the mechanical stability of FPSCs, maintaining 91% of the original efficiency after 10 000 bending cycles at a radius of 5 mm. Meanwhile, the unencapsulated 4‐HBA‐modified FPSCs exhibited outstanding environmental and light stability, retaining 82% of the original efficiency in an air environment with RH 20%‐30% and temperature of 25–30 °C for up to 1600 h, and maintaining 83% of its original efficiency under AM1.5 continuous irradiation for 1000 h. By comparing with a similar structure 3‐BA molecule in detail, it was concluded that the crosslinker 4‐HBA was more excellent and efficient for in situ polymerization‐controlled growth of perovskites and developing highly efficient and mechanically robust FPSCs.

## Conflict of Interest

The authors declare no conflict of interest.

## Supporting information



Supporting Information

Supporting Information

Supporting Information

## Data Availability

The data that support the findings of this study are available from the corresponding author upon reasonable request.
