# Supplementary material for: In Situ Polymerization Controlled Growth of Perovskite via Benzoic Acid Crosslinking Agent for Highly Efficient and Mechanically Robust Flexible Perovskite Solar Cell
Source: Adv Sci (Weinh). 2025 Jul 21;12(39):e08253. doi: 10.1002/advs.202508253 (PMC12533145; doi:10.1002/advs.202508253)
Supplement: Supplementary file 1 — Supporting Information [file ADVS-12-e08253-s002.docx]

**Supporting information**

**In Situ Polymerization Controlled Growth of Perovskites Based on Benzoic Acid Crosslinking Agent for Highly Efficient and Mechanically Robust** **Flexible Perovskite Solar Cell**

*Yankun Yang^a＃^, Yang Na ^a＃^, Chenxi Zhang^a^*, Shiqi Li ^a^,Yang Hao^a^, Qinjun Sun^a^, Zhihui Chen^a^*, Shengzhong (Frank) Liu^c^ , Fei Guo^d^, Jianfeng Lu^e^, Yuying Hao^a, b^**

^a^ *College of Physics and Optoelectronics Engineering, Shanxi Key Lab of Photovoltaic Technology and Application, Key Lab of Advanced Transducers and Intelligent Control System, Taiyuan University of Technology, Taiyuan 030024, China*

*^b^ Shanxi-Zheda Institute of Advanced Materials and Chemical Engineering, Taiyuan 030000.*

^c^ *Key Laboratory of Applied Surface and Colloid Chemistry, National Ministry of Education, Shaanxi Engineering Lab for Advanced Energy Technology, School of Materials Science and Engineering, Shaanxi Normal University, Xi’an 710119, China*

*^d^ Institute of New Energy Technology, College of Physics & Optoelectronic Engineering, Jinan University, Guangzhou 510632, China*

*^e^ State Key Laboratory of Silicate Materials for Architectures, Wuhan University of Technology, Wuhan 430070, China*

**Corresponding author. E-mail address: [zhangchenxi@tyut.edu.cn,](mailto:zhangchenxi@tyut.edu.cn,) haoyuying@tyut.edu.cn*

*^＃^ the authors made same contribution in this paper.*

**Materials and methods**

**Materials**

All materials and solvents included in this work were purchased from commercial suppliers and used without further purification. SnO_2_ colloidal precursor (tin (IV) oxide, 15% in H_2_O colloidal dispersion) was purchased from Alfa Aesar. 4-hydroxybenzoic acid and 3-benzoylpropionic acid was purchased from Shanghai Bide Pharmaceutical Company. Dimethylformamide (DMF), dimethyl sulfoxide (DMSO), chlorobenzene, toluene, and isopropyl alcohol were purchased from Sigma-Aldrich. Formamidinium iodide (FAI), methylammonium bromide (MABr) and methylammonium chloride (MACl), 2-phenethylamine hydroiodate (PEAI), Bistrifluoromethane sulfonimide Lithiumwere (LiTFSI), 4-tert-Butylpyridine (TBP) were all purchased from Xi’an Polymer Light Technology in China. Lead iodide (PbI_2_) was purchased from TCI Company. Spiro-OMeTAD was purchased from Liaoning Preferred New Energy Technology in China.

**Device fabrication**

**Rigid devices**

The ITO glasses were cleaned sequentially by deionized water, acetone and isopropanol (IPA) for 15 minutes, respectively. Before use, the ITO glasses were treated with UV-ozone for 15 minutes. Then SnO_2_ precursor (SnO_2_ colloidal: deionized water = 1: 4) was spin-coated on the ITO substrate at 4000 rpm for 30 s and annealed in ambient air at 150℃ for 30 min. After that, 1.3M PbI_2_ precursor in DMF: DMSO (19: 1) solvent was spin-coated onto SnO_2_ layer at 1500 rpm. for 30 s, then annealed at 70 ℃ for 1 min, and then cooled to room temperature. For FA_x_MA_1-x_PbI_3_ perovskite films, an organic ammonium salt solution consisting of FAI, MABr, and MACl (60 mg: 6 mg: 6 mg in 1 mL IPA) was first prepared. Subsequently, 0.4 mg/mL of 3-BA and 4-HBA were incorporated into the organic ammonium salt solution. The resulting solution was spin-coated onto lead iodide at the rotational rate of 1700 rpm for 30 s, followed by thermal annealing at 115 °C for 25 min in ambient air. After perovskite film formation, the samples were transferred to a nitrogen-filled glove box for further processing. Subsequently, 5 mg/ml PEAI was spun onto the perovskite active layer at a rotational rate of 5000 rpm for 30 s. Afterwards, Spiro-OMeTAD solution, that perpared by dissolving 72.3 mg Spiro-OMeTAD, 17.5 μL Li–TFSI (520 mg/mL in acetonitrile), and 28 μL TBP in 1 mL chlorobenzene, was spin-coated onto the perovskite film at the rate of 4000 rpm for 30 s. Finally, the 110 nm-thick Ag electrode was deposited by thermal evaporation under a high vacuum.

**Flexible devices**

The PET/ITO was cleaned by sonication in detergents/H_2_O, deionized water and IPA for 15 min sequentially. Before use, the PET/ITO was treated with UV-ozone for 15 minutes. Subsequently, the SnO_2_ film and perovskite films were prepared in the same way as the rigid devices, except that the annealing temperature was 100°C. The preparation methods of PEAI thin film, Spiro-OMeTAD hole transport layer and Ag electrode also were the same as those of rigid devices.

**Characterization**

The optical transmittance spectra and absorption spectra were measured using a Shimadzu UV-2600. The GIXRD plots were taken Bruker+D8 Advance to test. The XRD patterns were taken on a Bruker QUANTAX 200 diffractometer. XPS were carried out using a photoelectron spectrometer (ESCALAB250Xi, Thermo Fisher Scientific). SnO_2_ nanoparticle size and zeta potential were measured by a Malvern Zetasizer Nano ZS9. AFM was performed using an NX10 atomic force microscope from Park Systems. The surface morphology of the thin films was characterized by SEM (JEOL JSM-6340, Japan). The steady-state PL spectra and TRPL spectra were measured by using a fluorescence spectrometer (FLS980, Edinburgh Instruments). The fourier transform infrared (FTIR), the nuclear magnetic hydrogen spectra (^1^H-NMR) and the matrix assisted laser desorption/ionization time-of-flight mass spectrometry (MALDI-TOF ) were measured by TENSOR 27, AVANCE III HD 600MHz and Bruker autoflex speed MALDI TOF, respectively, to inveatigate the polymerization of 4-HBA. For MALDI-TOF spectra, the formula X_n_= R_P_/R_t_ l∑R_tr_ is commonly employed to calculate the degree of aggregation of polymer. R_P_ is the number of monomers and R_t_ l∑R_tr_ is the number of macromolecules.

A solar simulator (ABET SUN3000) was used to provide simulated solar irradiation (AM1.5G,100mW/cm^2^). The measurement of *J*–*V* characteristics and the MPP test were performed using a Keithley 2400 source meter. The output of the light source was adjusted using a calibrated silicon photodiode (ABET Technology). The EQE was measured using a power source (ZOLIX CSC1011) with a monochromator and a source meter. The UPS was used to characterize the energy levels (Therom Fisher Scientific ESCALAB Xi+). The TPV and TPC were used to test carrier transport, accumulation, recombination, and other processes (TranPVC).


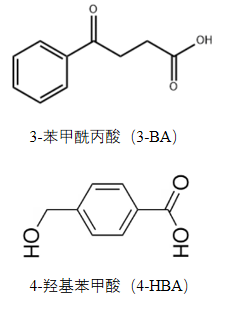


**Figure S1.**Structural formula of 3-BA

**
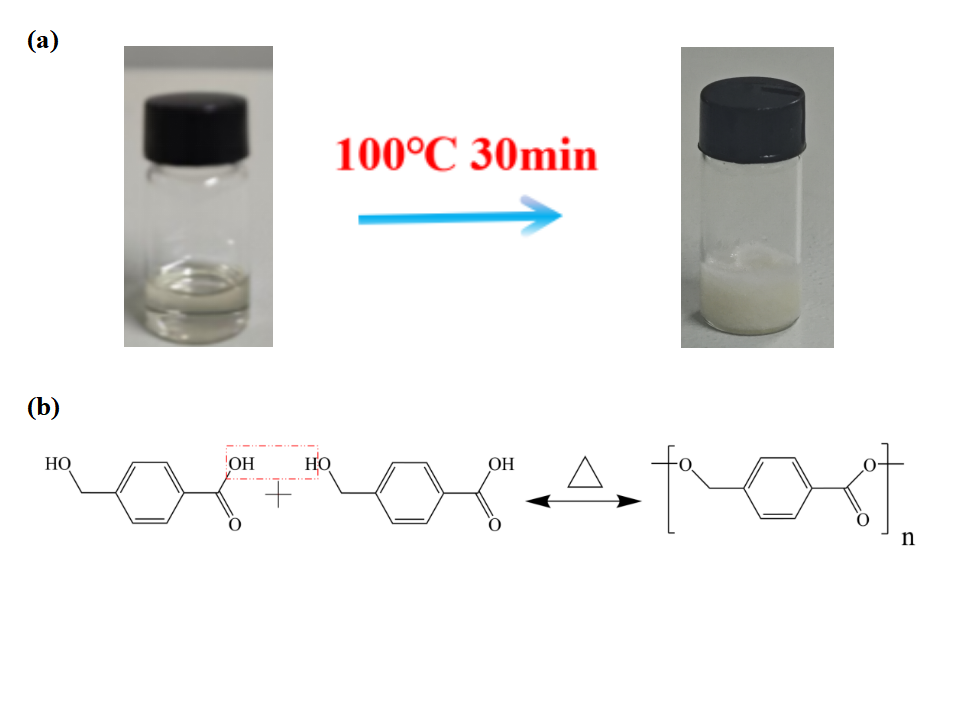
**

**Figure S2.** Photos of 4-HBA before and after cross-linking


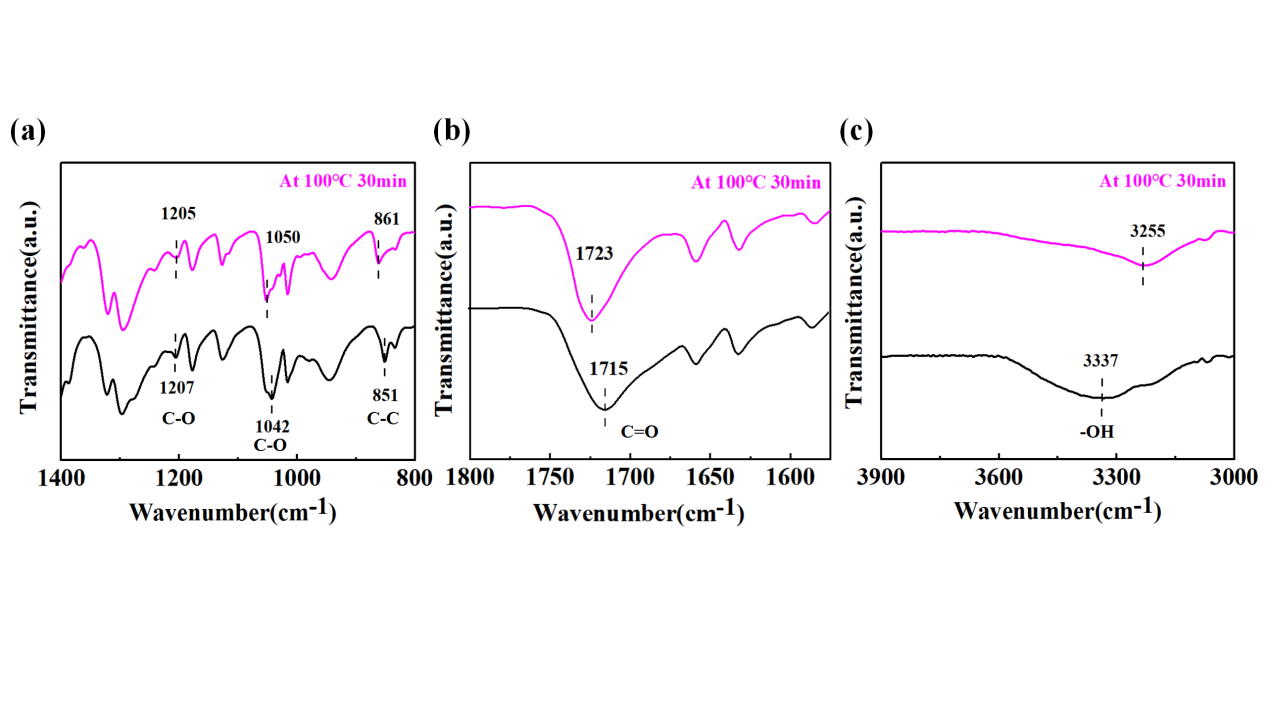


**Figure S3.** FTIR spectra of 4-HBA before and after cross-linking


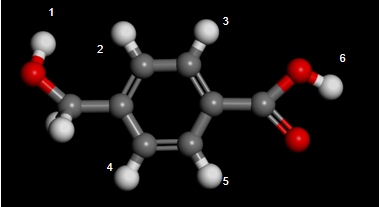

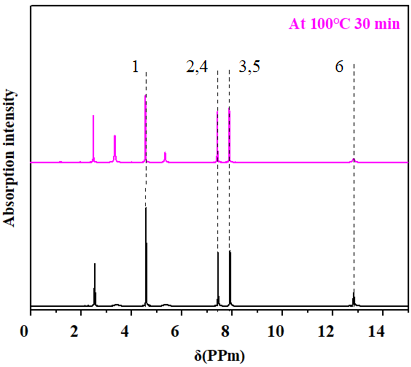


**Figure S4.** Nuclear magnetic hydrogen spectra of 4-HBA before and after crosslinking

**Table S1.** Statistics of chemical shift parameters of 4-HBA nuclear magnetic hydrogen spectrum after polymerization treatment

|  | Position of hydrogen atom | Chemical displacement | Functional group |
| --- | --- | --- | --- |
| （4-HBA） | 2,4 | 7.42 7.44 | Hydrogen of benzene ring Ha |
|  | 3,5 | 7.89 7.91 | Hydrogen of benzene ring Hb |
|  | 1 | 4.58 | Hydroxyl group（-OH） |
|  | 6 | 12.83 | Carboxyl group（-COOH) |
| （4 - HBA polymerization） | 2,4 | 7.41 7.43 | Hydrogen of benzene ring Ha |
|  | 3,5 | 7.90 7.92 | Hydrogen of benzene ring Hb |
|  | 1 | 4.53 | Hydroxyl group（-OH） |
|  | 6 | 12.83 | Carboxyl group（-COOH) |

**Table S2.** Statistical Summary of Integral Area Ratio Parameters of 4-HBA Before and After Aggregation Treatment in ¹H NMR Spectra

|  |  | Integral area | Functional group | Integral ratio（I_1_/I_2_) |
| --- | --- | --- | --- | --- |
| 4-HBA | I(4.58) | 74366 | Hydroxyl group | [I(4.58)-I(4.53)]/I(4.58）=0.273 |
| （4 - HBA polymerization） | I(4.53) | 54091 | Hydroxyl group |  |


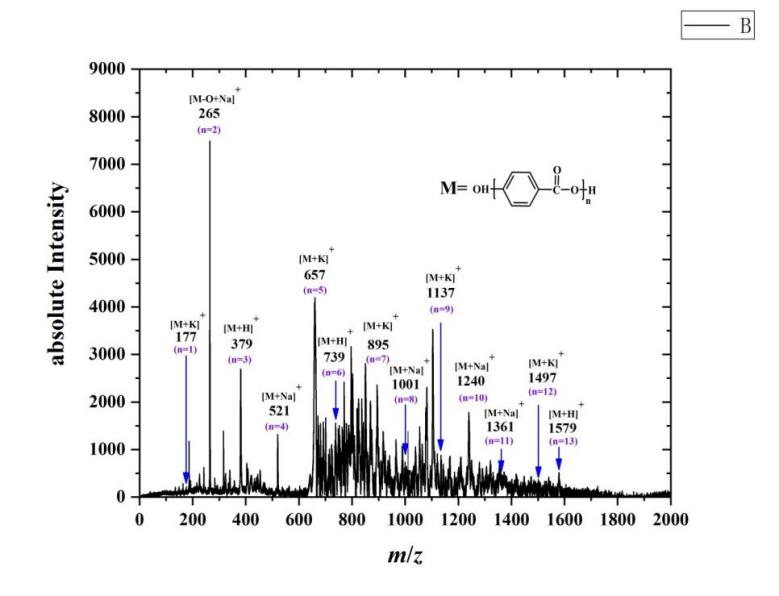


**Figure S5.** MALDI mass spectra of poly-p-hydroxybenzoic acid

**Table S3.** Structure of 4HBA chain ends as deduced from the mass determined from the MALDI-TOF spectrum

| **Series** | ***M/z*** | **Quantity of n** | | **Structure** |
| --- | --- | --- | --- | --- |
| 1 | 177 | | 1 | 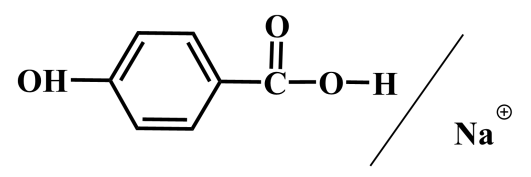 |
| 2 | 265 | | 2 | 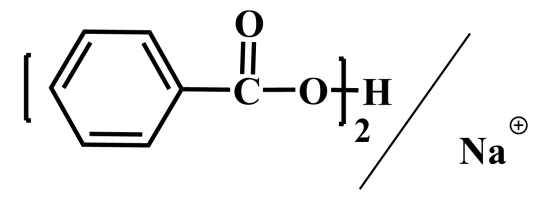 |
| 3 | 397 | | 3 | 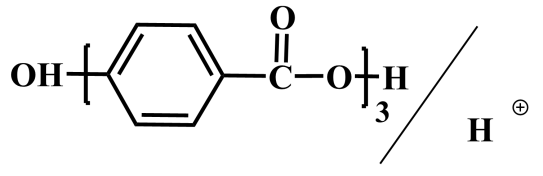 |
| 4 | 521 | | 4 | 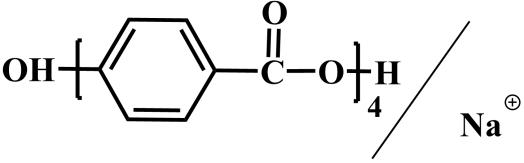 |
| 5 | 657 | | 5 | 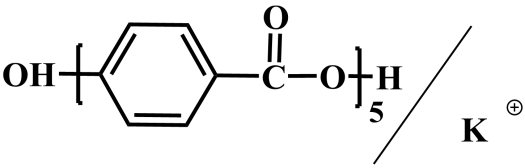 |
| 6 | 739 | | 6 | 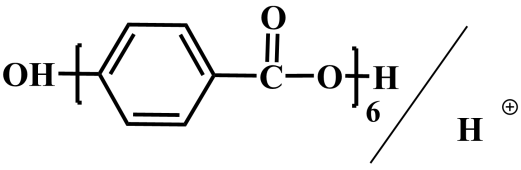 |
| 7 | 895 | | 7 | 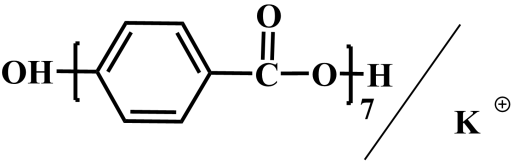 |
| 8 | 1001 | | 8 | 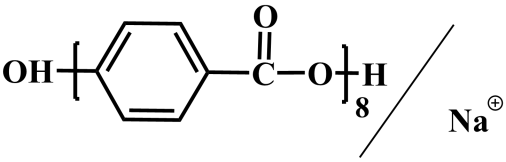 |
| 9 | 1137 | | 9 | 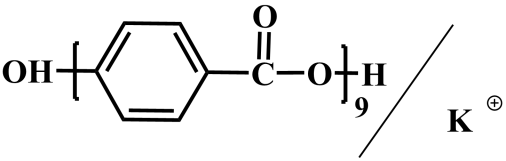 |
| 10 | 1240 | | 10 | 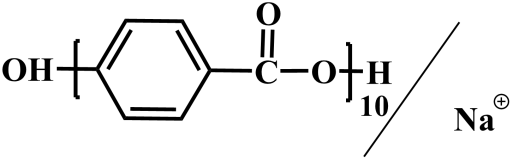 |
| 11 | 1361 | | 11 | 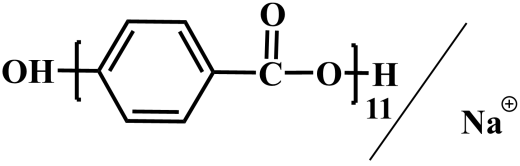 |
| 12 | 1497 | | 12 | 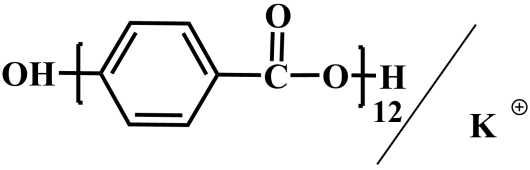 |
| 13 | 1579 | | 13 | 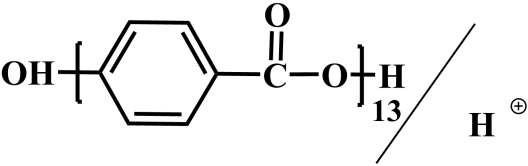 |


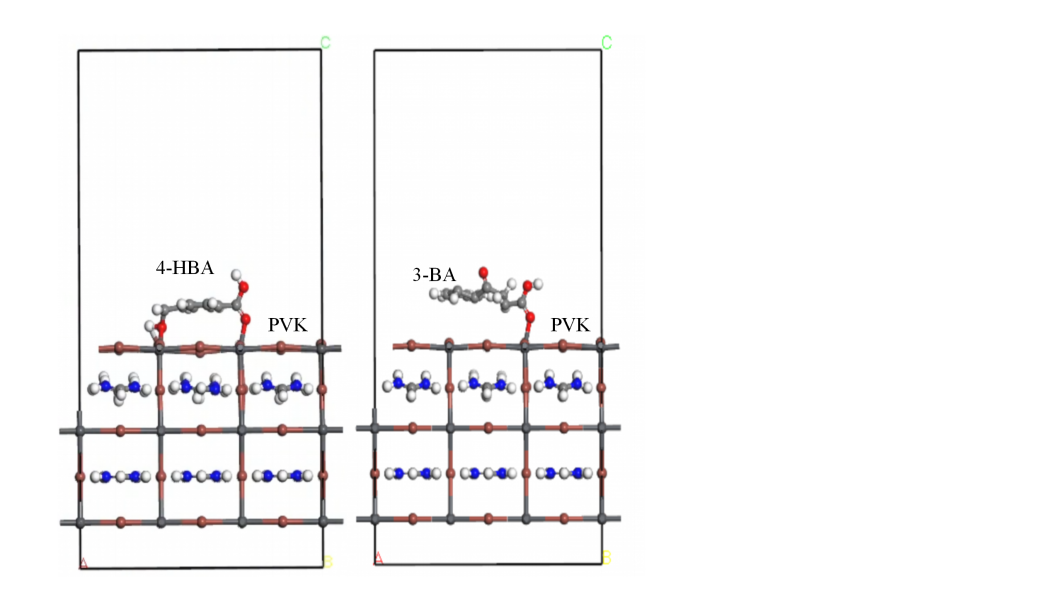


**Figure S6.** Molecular models of 4-HBA-PVK and 3-BA-PVK

**Table S4.** Binding energy parameter statistics of 4-HBA-PVK and 3-BA-PVK

|  | Total Energy (eV) | Binding energy (eV) |
| --- | --- | --- |
| 4-HBA-PVK | -8.67E+02 | 11.69 |
| 3-BA-PVK | -8.83E+02 | 4.57 |

**
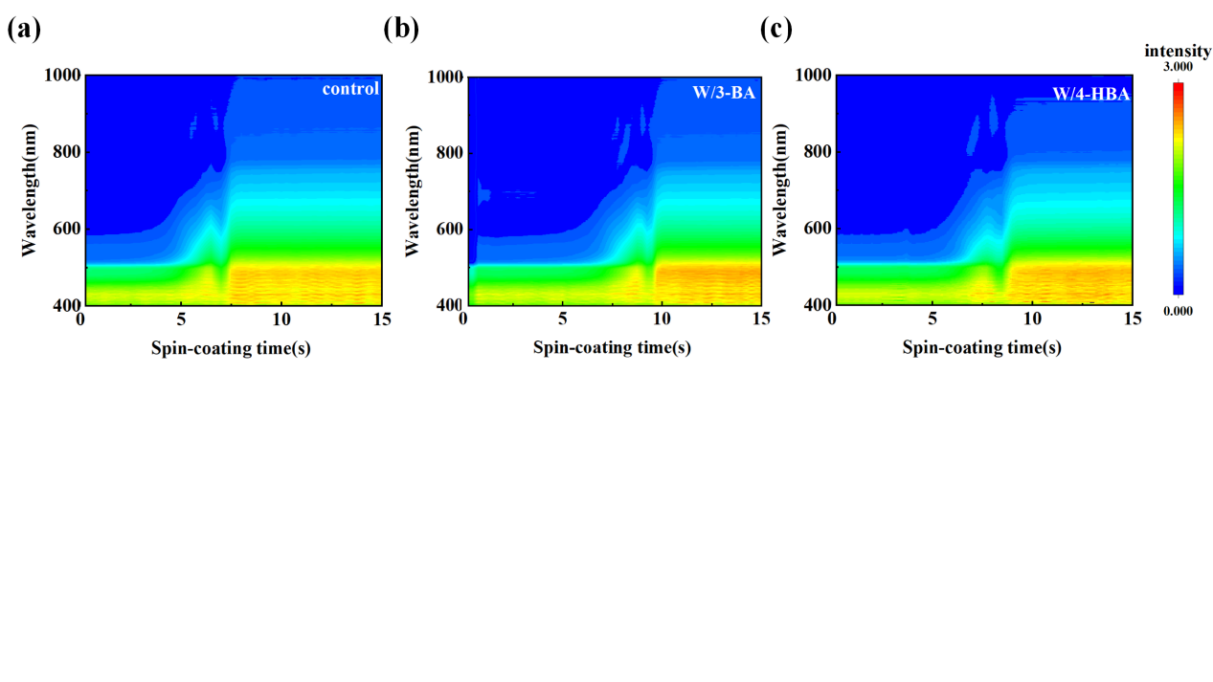
**

**Figure S7.** In situ UV–vis absorption spectra of perovskite wet film during spin-coating of organic ammonium salt on PbI_2_ film


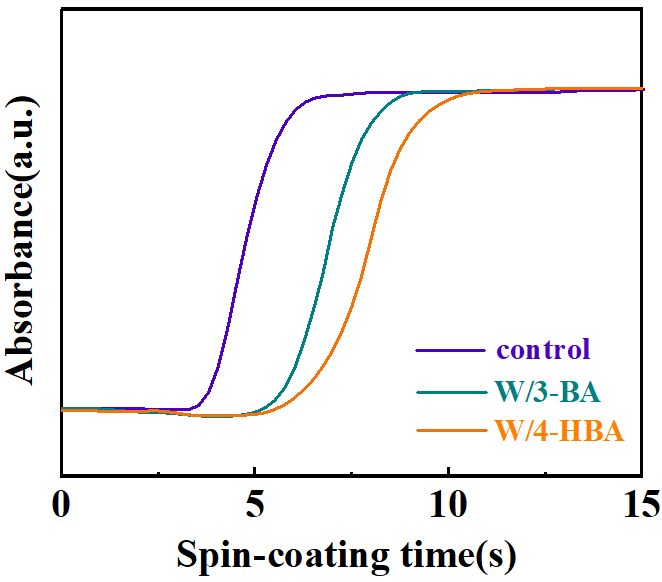


**Figure S8.** The absorption intensity (AI) evolution with time at 700 nm extracted from Figure S6


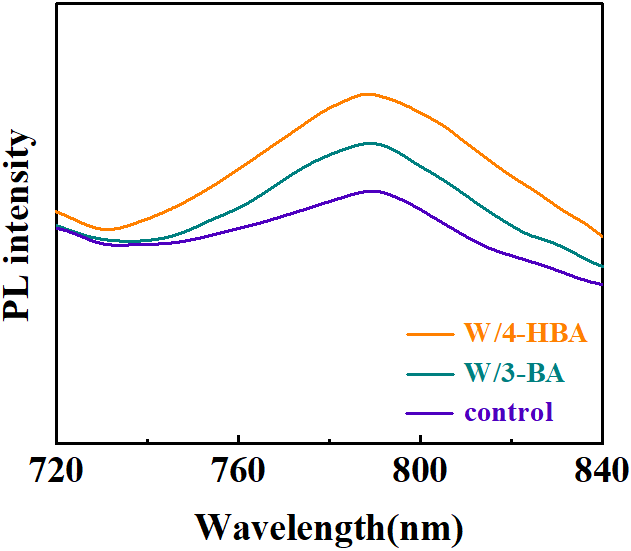


**Figure S9.** PL spectra of perovskite wet film when annealed for 10 s extracted from Figure 3d-f

**Table S5.** The carrier lifetime statistics of different perovskite films extracted from transient photoluminescence spectra (TRPL)

|  | Λ1 | *τ*_1_(s) | Λ_2_ | *τ*_2_(s) | *t_a_*_ve_(s) |
| --- | --- | --- | --- | --- | --- |
| control | 22.88 | 888.03 | 65.19 | 2451.37 | 0.753 |
| 3-BA | 40.76 | 1117.85 | 75.56 | 2656.34 | 1.036 |
| 4-HBA | 43.18 | 1209.09 | 68.30 | 2871.04 | 1.147 |


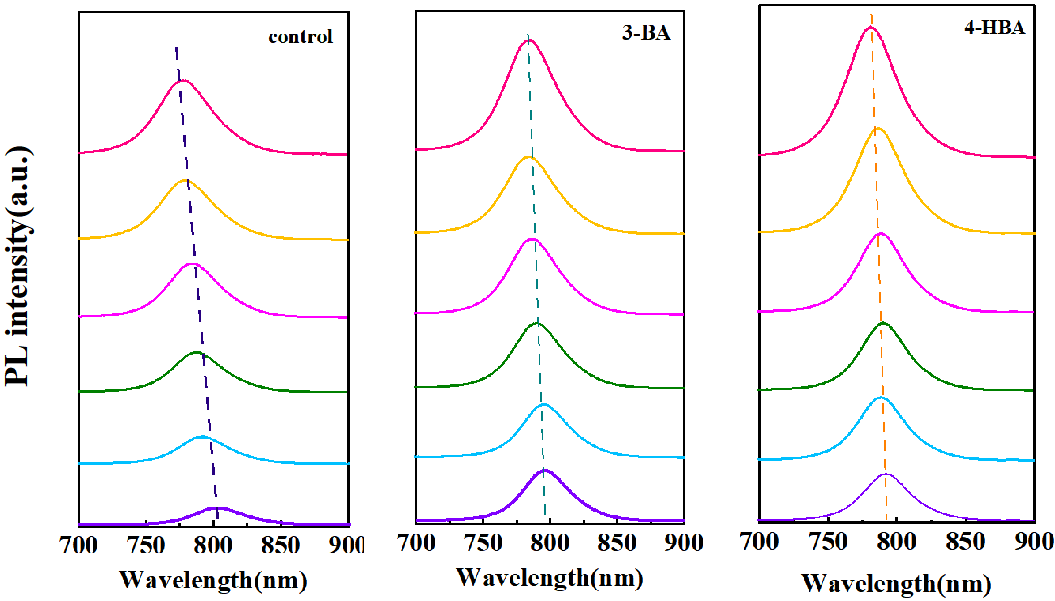


**Figure S10.** Evolution of steady-state PL spectra of different perovskite films at different pressures


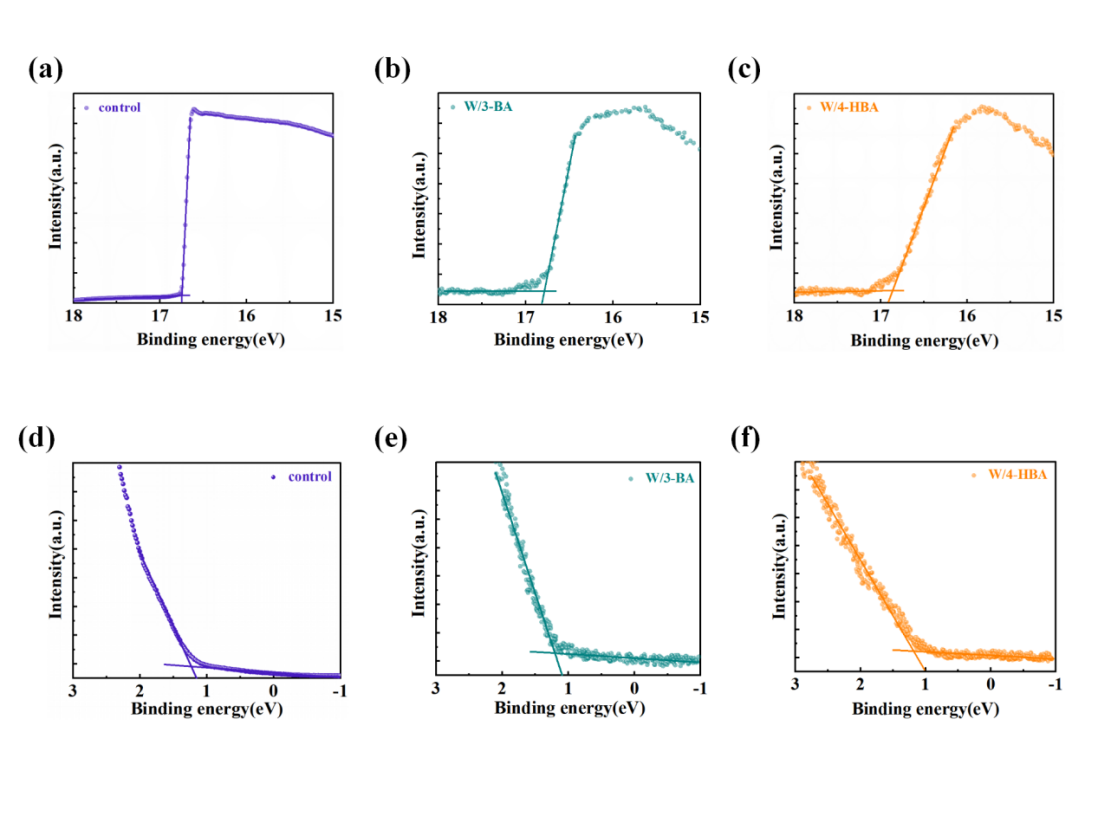


**Figure S11.** Ultraviolet photoelectron spectroscopy (UPS) characterization of different perovskite films


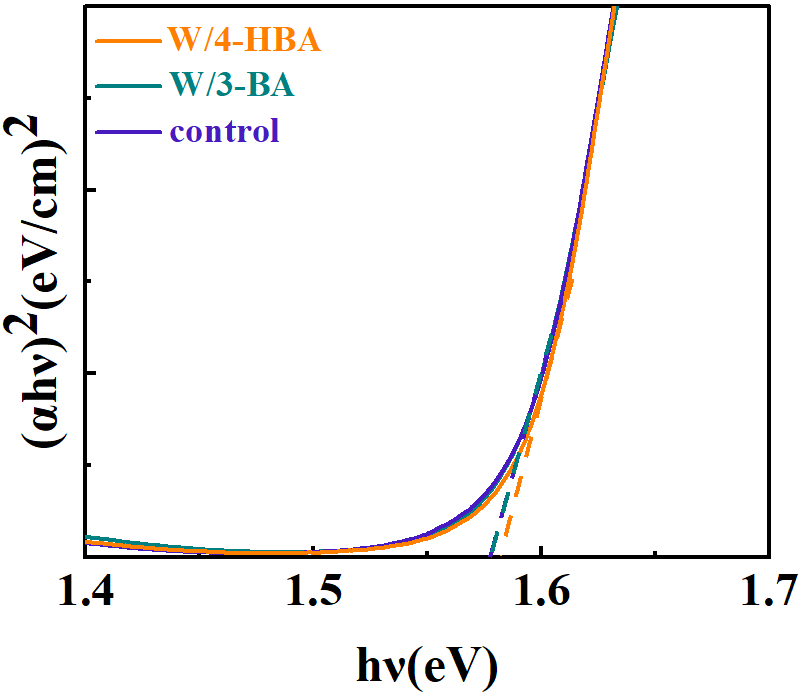


**Figure S12.** Tauc plots of the different perovskite films


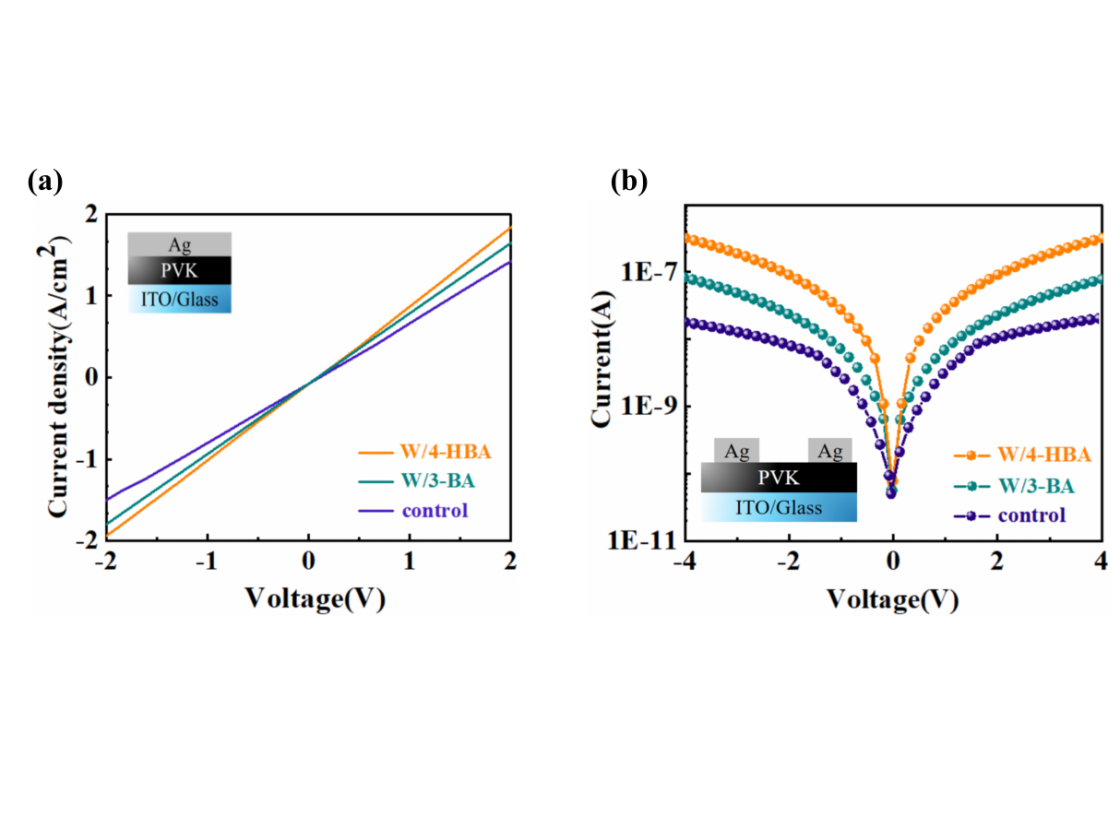


**Figure S13.** Longitudinal conductivity of different perovskite film


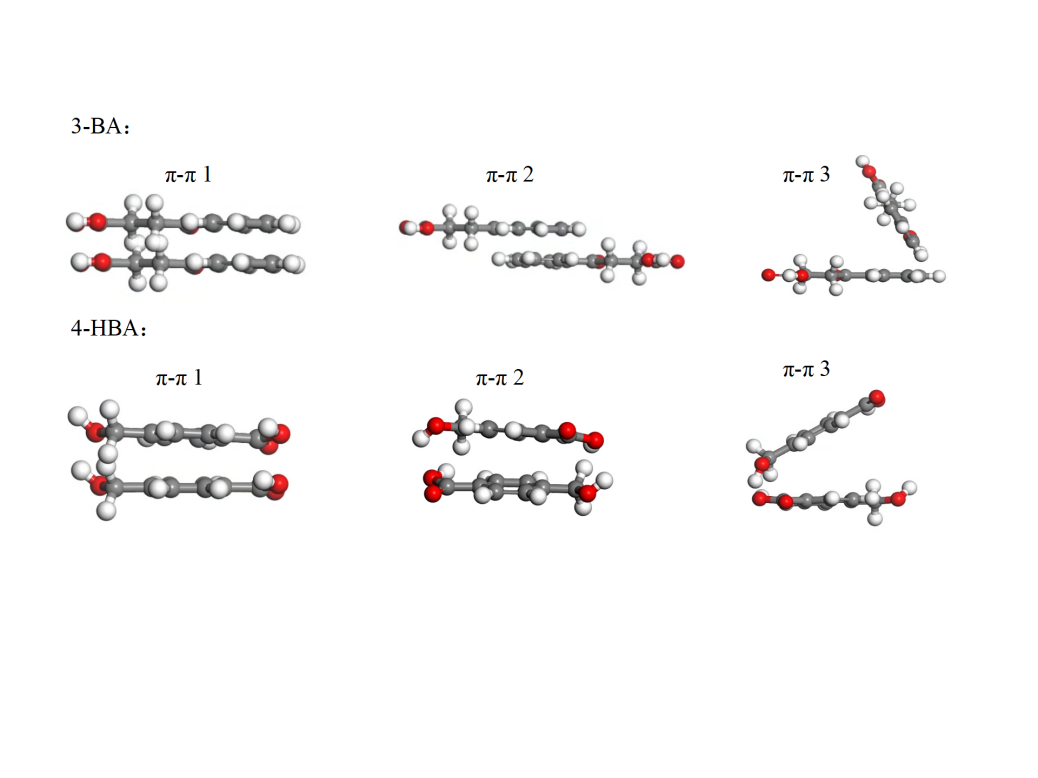


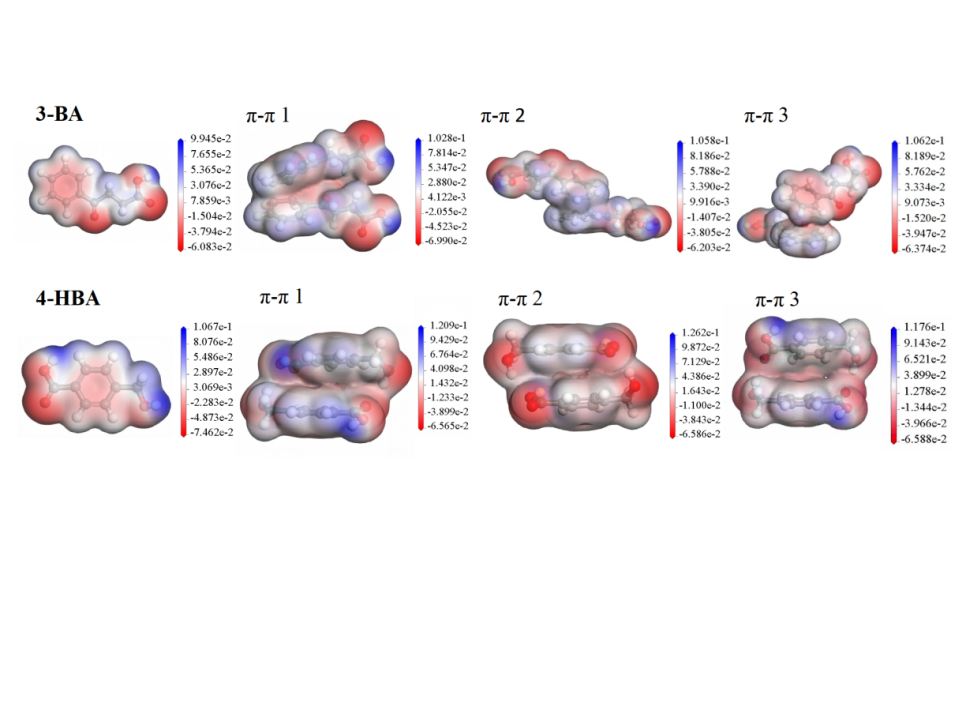


**Figure S14.** The original configuration of π-π interaction for 3-BA and 4-HBA, among them, π-π1 represents the intermolecular parallel configuration; π-π2 represents a 180° intermolecular configuration; π-π3 represents a 45° intermolecular configuration (upper); The optimized molecular configuration and electrostatic potential distribution (ESP) of 3-BA and 4-HBA (under).

**Table S6.** Statistics of interaction parameters between 4-HBA molecules and 3-BA molecules

|  | π-π model | Binding energy | π-π interaction energy (molecular-molecular) |
| --- | --- | --- | --- |
| 4-HBA | π-π 1 | 2.17 | 0.25 |
|  | π-π 2 | 2.21 | 0.25 |
|  | π-π 3 | 2.28 | 0.19 |
| 3-BA | π-π 1 | 0.23 | 0.00238 |
|  | π-π 2 | 0.52 | 0.00 |
|  | π-π 3 | 1.72 | 0.17 |

**Table S7.** photovoltaic parameters of Rigid PSCs

|  | Dosage concentration | Jsc [mA cm^−2^] | Voc [V] | FF | PCE [%] average | PCE [%] best |
| --- | --- | --- | --- | --- | --- | --- |
| control | -- | 23.76±0.35 | 1.169±0.079 | 0.754±0.015 | 21.41±0.57 | 22.32 |
| Device  W/4-HBA | 0.2mg/ml | 25.07±0.39 | 1.179±0.033 | 0.788±0.007 | 23.14±0.48 | 24.19 |
|  | 0.4mg/ml | 25.08±0.21 | 1.185±0.029 | 0.794±0.006 | 23.94±0.32 | **24.76** |
|  | 0.6mg/ml | 25.02±0.34 | 1.181±0.032 | 0.785±0.008 | 23.21±0.42 | 23.94 |
|  | 0.8mg/ml | 24.67±0.56 | 1.156±0.044 | 0.769±0.010 | 22.28±0.53 | 22.72 |
| Device  W/3-BA | 0.2mg/ml | 24.44±0.40 | 1.175±0.038 | 0.776±0.018 | 22.91±0.49 | 23.05 |
|  | 0.4mg/ml | 24.92±0.28 | 1.183±0.029 | 0.782±0.014 | 23.18±0.35 | **23.83** |
|  | 0.6mg/ml | 24.41±0.42 | 1.177±0.036 | 0.780±0.016 | 23.10±0.51 | 23.56 |
|  | 0.8mg/ml | 24.12±0.58 | 1.151±0.048 | 0.766±0.019 | 22.15±0.62 | 22.66 |


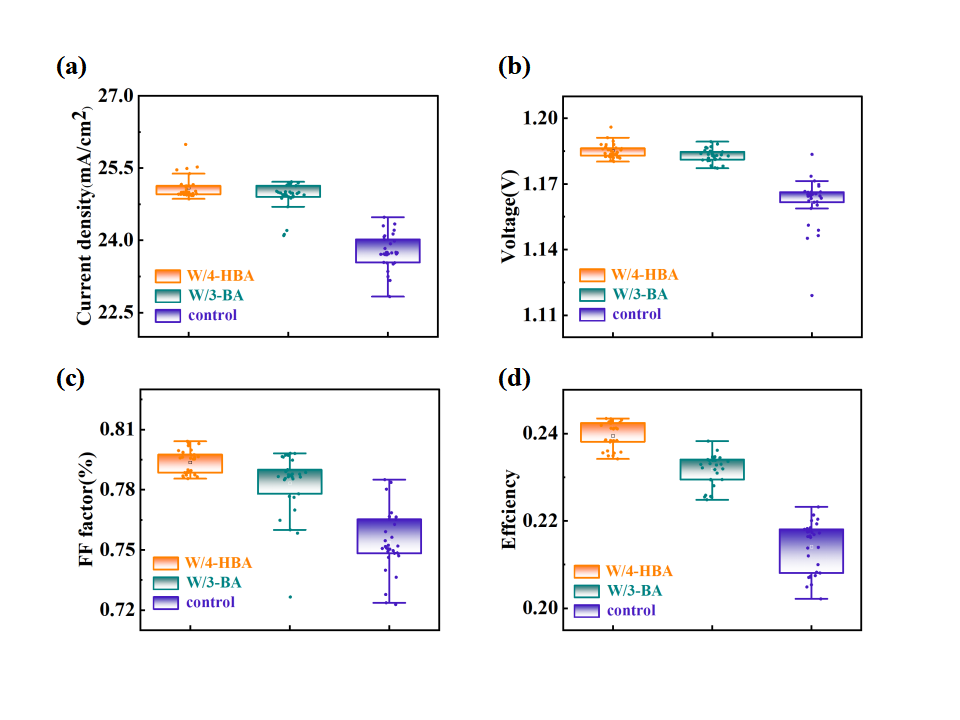


**Figure S15.** Photovoltaic parameter statistics of 30 rigid PSCs

**Table S8.** photovoltaic parameters of FPSCs

| Flexible device | Jsc [mA cm^−2^] | Voc [V] | FF | PCE [%] average | PCE [%] best |
| --- | --- | --- | --- | --- | --- |
| control | 22.59±0.46 | 1.157±0.105 | 0.773±0.013 | 20.00±0.37 | 20.69 |
| W/3-BA | 22.93±0.27 | 1.178±0.076 | 0.788±0.008 | 21.34±0.29 | 21.78 |
| W/4-HBA | 23.22±0.16 | 1.184±0.041 | 0.794±0.005 | 22.42±0.27 | 22.73 |


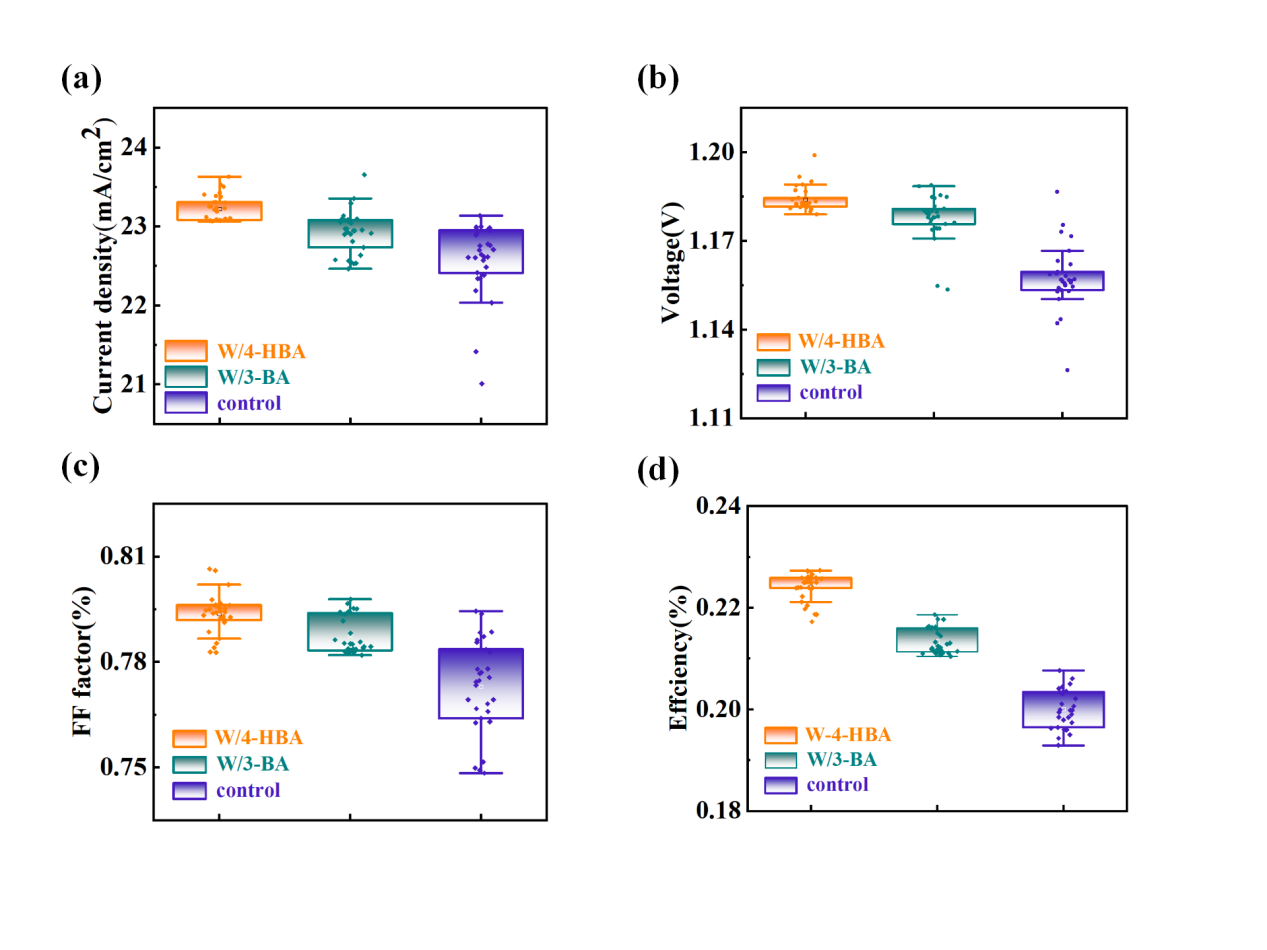


**Figure S16.** Photovoltaic parameter statistics of 30 FPSCs


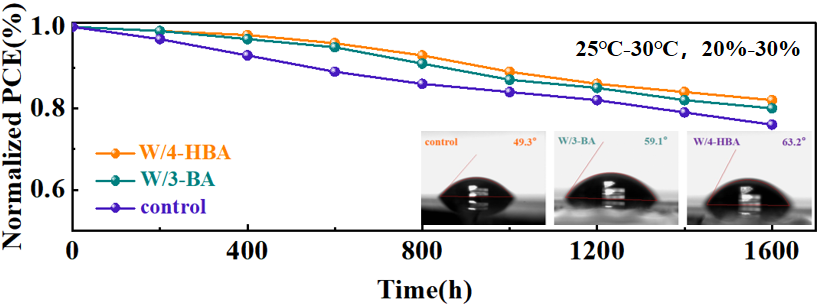


**Figure S17.** Moisture stability test of FPSCs in ambient air with the relative humidity (RH) of 20-30% and the temperature of 25℃ -30℃ and the water contact angle test of perovskite film


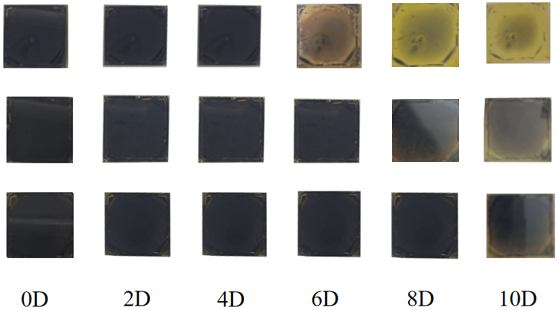


**Figure S18.** Stability of perovskite film stored for 10 day in ambient air with RH 60% -70% and the temperature of 25℃ -30℃


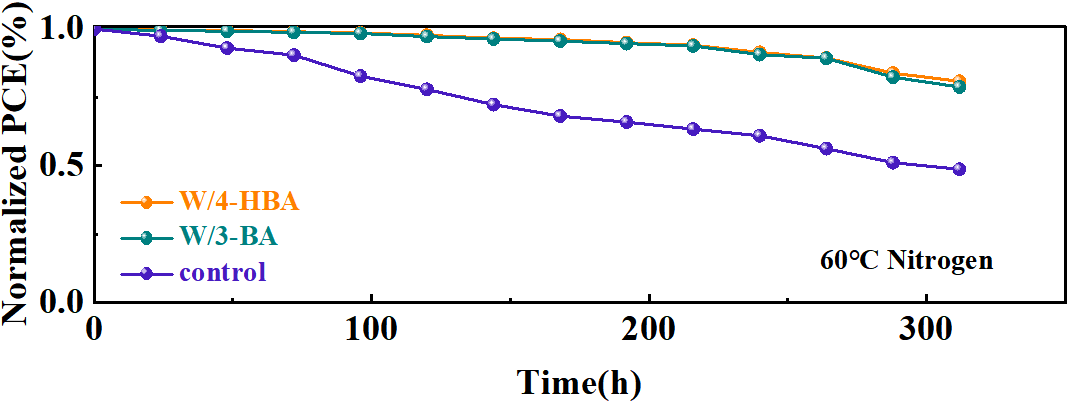


**Figure S19.** Thermal stability of the unsealed FPSCs stroed in the nitrogen-filled glovebox at 60 °C in the dark
